# Adipocyte caspase-8 but not RIPK3 promotes adiposity

**DOI:** 10.1038/s41420-026-03201-z

**Published:** 2026-06-23

**Authors:** Carmen K. Chan, Rukhsana Aslam, Fan Yang, Arshdeep Panesar, Anne Hakem, Razqallah Hakem, Herbert Y. Gaisano, Daniel A. Winer, Cynthia T. Luk

**Affiliations:** 1 Keenan Research Centre for Biomedical Science, St. Michael’s Hospital, Unity Health Toronto, Toronto, ON Canada; 2 Institute of Medical Science, Temerty Faculty of Medicine, University of Toronto, Toronto, ON Canada; 3 Princess Margaret Cancer Centre, University Health Network, Toronto, ON Canada; 4 Department of Laboratory Medicine and Pathobiology, University of Toronto, Toronto, ON Canada; 5 Department of Medical Biophysics, University of Toronto, Toronto, ON Canada; 6 Toronto General Hospital Research Institute, University Health Network, Toronto, ON Canada; 7 Department of Medicine, Temerty Faculty of Medicine, University of Toronto, Toronto, ON Canada; 8 Banting and Best Diabetes Centre, University of Toronto, Toronto, ON Canada; 9 Department of Immunology, University of Toronto, Toronto, ON Canada; 10 Buck Institute for Research on Aging, Novato, CA USA; 11 Division of Endocrinology and Metabolism, Department of Medicine, St. Michael’s Hospital, Unity Health Toronto, Toronto, ON Canada

**Keywords:** Mechanisms of disease, Necroptosis, Metabolic syndrome

## Abstract

Adipocyte death is a key event in the development of white adipose tissue (WAT) inflammation, a major driver of obesity-associated metabolic dysfunction. Receptor-interacting protein kinase 3 (RIPK3) mediates necroptosis, a recently discovered mode of regulated necrosis. Necroptosis has been implicated in several inflammatory pathologies; however, the role of adipocyte necroptosis in obesity remains unclear. In the present study, we sought to investigate the role of adipocyte RIPK3 in obesity and glucose homeostasis. We demonstrated that necroptotic signalling was upregulated in WAT of mice with diet-induced obesity and was associated with body-mass index in human WAT. We also demonstrated that caspase-8, a central regulator of apoptosis, suppresses adipocyte necroptosis both in vitro and in vivo. Adipocyte-specific deletion of caspase-8 in mice reduced adiposity compared to control mice. This difference was not observed with concomitant global deletion of RIPK3. Furthermore, adipocyte-specific deletion of the RIPK3 receptor-interacting protein homotypic interaction motif (RHIM), which is required for necroptotic induction, did not influence weight gain, adiposity, or glucose homeostasis in mice with diet-induced obesity. *Caspase-8* knockdown by siRNA or pharmacological inhibition in 3T3-L1 adipocytes suppressed adipogenesis, which may be independent of adipocyte *Ripk3*. Collectively, our findings suggest that adipocyte RIPK3 RHIM does not play a critical role in obesity and glucose homeostasis. Alternatively, we provide further evidence that caspase-8 plays an essential role in adipocyte differentiation, offering insight into the molecular mechanisms underlying obesity and metabolic dysfunction.

## Introduction

Obesity is estimated to have doubled among women and tripled among men globally between 1990 and 2022 [1]. Heightened morbidity and mortality in individuals with overweight and obesity [2] are largely attributed to increased risk of metabolic dysfunction, including insulin resistance and cardiovascular diseases [2, 3]. Consequently, obesity and associated metabolic dysfunction poses an extensive healthcare and economic burden [4, 5].

A pro-inflammatory shift in white adipose tissue (WAT) during obesity is suggested to be a major driver of obesity-associated metabolic dysfunction [6–8]. WAT inflammation, characterized by macrophage infiltration and pro-inflammatory cytokine release [6, 7, 9–14], was reported to disrupt insulin signalling [14, 15]; driving systemic insulin resistance. Consistently, reducing WAT inflammation improves glucose homeostasis in obese mice [13, 14, 16].

Adipocyte death with necrosis-like morphology is described as an important event in the development of WAT inflammation [6]. Necroptosis is an inflammatory mode of necrotic cell death [17] implicated in several human pathologies with immune components, including metabolic dysfunction-associated steatotic liver disease [18–22] and cardiovascular disease [23, 24]. Thus, defining the role of adipocyte necroptosis may offer insight into the mechanisms underlying obesity, WAT inflammation, and metabolic dysfunction.

Receptor-interacting protein kinase 3 (RIPK3) is a key mediator of necroptosis that phosphorylates necroptotic effector mixed lineage domain-like pseudokinase (MLKL) [25]. Oligomerization of phosphorylated MLKL facilitates membrane permeabilization and leakage of pro-inflammatory intracellular contents [25, 26]. In line with the pro-inflammatory nature of necroptosis, global deletion of MLKL improved glucose homeostasis in obese mice [20]. Unexpectedly, global deletion of RIPK3 in obese mice worsened WAT inflammation and glucose homeostasis by exacerbating caspase-8-mediated adipocyte apoptosis [27], which is in line with other reports of RIPK3 limiting caspase-8-mediated apoptosis [28, 29]. Consistent with these findings, our group previously demonstrated that adipocyte-specific disruption of caspase-8 reduced adiposity and improved WAT inflammation and glucose homeostasis in obese mice [30]. Collectively, the role of adipocyte necroptosis and crosstalk between caspase-8 and RIPK3 in the pathogenesis of obesity and metabolic dysfunction remains elusive.

We sought to further clarify the role of adipocyte RIPK3 and its necroptotic function in obesity and glucose homeostasis using adipocyte-specific methods. We find that RIPK3 and necroptotic signalling are upregulated in WAT. We demonstrate that disruption of caspase-8 activates necroptotic signalling in adipocytes. Adipocyte-specific disruption of caspase-8 reduces adiposity in mice with diet-induced obesity; however, this difference is not observed with concomitant global deletion of RIPK3. Furthermore, adipocyte-specific loss of the RIPK3 receptor-interacting protein homotypic interaction motif (RHIM), which is required for necroptotic induction, does not alter weight gain, adiposity, or glucose homeostasis in mice with diet-induced obesity. We then explore crosstalk of caspase-8 and RIPK3 in adipocytes, demonstrating that caspase-8 regulates adipogenesis in vitro, though this process may be independent of RIPK3. Our findings demonstrate that the RIPK3 RHIM is dispensable in adipocytes for obesity and loss of glucose homeostasis, and reveal a novel, non-apoptotic function of caspase-8 in adipocytes that may drive WAT expansion, contributing to obesity and metabolic dysfunction.

## Results

### Diet-induced obesity increases RIPK3 and necroptotic signalling in white adipose tissue

**Fig. 1:**
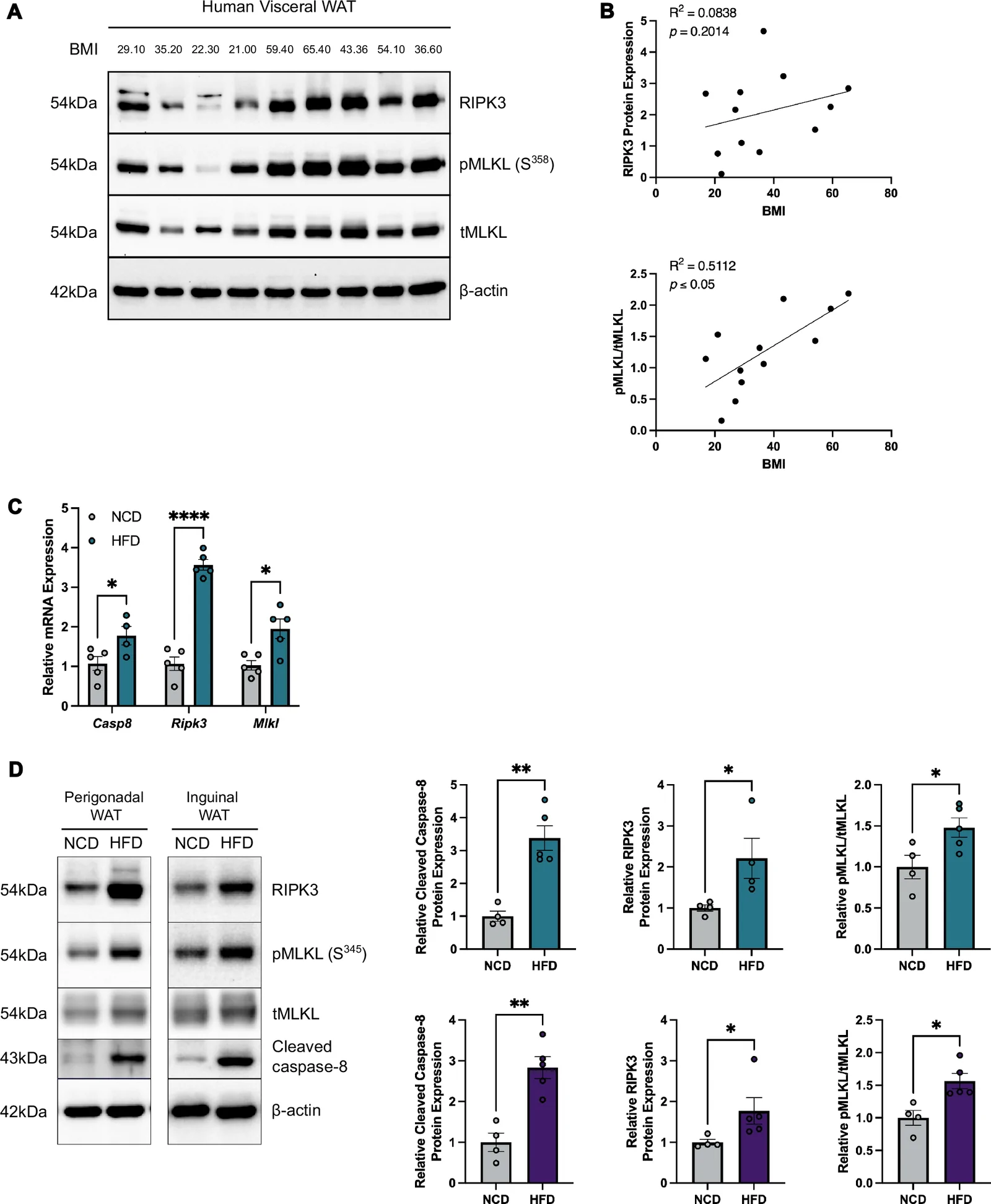
Diet-induced obesity increases RIPK3 and necroptotic signalling in white adipose tissue. **A** Immunoblot of human RIPK3, phosphorylated MLKL at S^358^ (pMLKL), and total MLKL (tMLKL) in human visceral WAT and body-mass index (BMI) of each subject. β-actin as loading control. *n* = 12 subjects. **B** Simple linear regression between BMI and human RIPK3 protein expression (top) or ratio of pMLKL to tMLKL (bottom). **C** Relative murine *Casp8*, *Ripk3*, and *Mlkl* mRNA expression in whole perigonadal WAT from male mice fed NCD (*n* = 5) or HFD (*n* = 4–5) for 16 weeks. *Rplp0* as endogenous control. **D** Immunoblot (left) and quantification (right) of murine RIPK3, pMLKL at S^345^, tMLKL, and cleaved caspase-8 in whole perigonadal (top) and inguinal (bottom) WAT of male mice fed NCD (*n* = 4–5) or HFD (*n* = 5) for 12 weeks. β-actin as loading control. Data are mean ± SEM. **C** * *p* ≤ 0.05 or **** *p* ≤ 0.0001 by unpaired Student’s *t* test with Holm-Šídák correction for multiple comparisons. **D** * *p* ≤ 0.05 by unpaired Student’s *t* test or Mann-Whitney *U*-test (RIPK3 in inguinal WAT). Non-specific binding (NSB). **A**, **D** β-actin shown is representative of multiple blots (See original western blots in Supplementary Information).

Necroptosis is an inflammatory mode of cell death characterized by phosphorylation of serine-358 (S^358^) of human MLKL or phosphorylation of serine-345 (S^345^) of murine MLKL. Phosphorylation of S^358^ on human MLKL, but not RIPK3 protein expression, in human visceral WAT demonstrated a positive trend with body-mass index (Fig. 1A, B; Supplementary Fig. S1 and Supplementary Table ST1), which is consistent with work by Gautheron and colleagues [27]. We also examined changes in adipose tissue RIPK3 and necroptotic signalling using six-week-old male C57BL/6 mice fed a normal chow diet (NCD) or a high-fat diet (HFD) for 16 weeks as a model of diet-induced obesity. *Casp8*, *Ripk3*, and *Mlkl* mRNA expression were all significantly increased in perigonadal WAT of mice with diet-induced obesity (Fig. 1C). Consistent with increased mRNA expression, RIPK3 protein expression and phosphorylation of MLKL were also significantly increased in both perigonadal and inguinal WAT of mice with diet-induced obesity compared to lean controls (Fig. 1D). Similarly, cleaved caspase-8 expression was significantly increased in both perigonadal WAT, which was also previously shown by our group in a separate experiment [30], and inguinal WAT of mice with diet-induced obesity compared to lean controls (Fig. 1D). These findings demonstrate an upregulation of RIPK3 and necroptotic signalling in WAT during diet-induced obesity.

### Disruption of caspase-8 activates necroptotic signalling in adipocytes

**Fig. 2:**
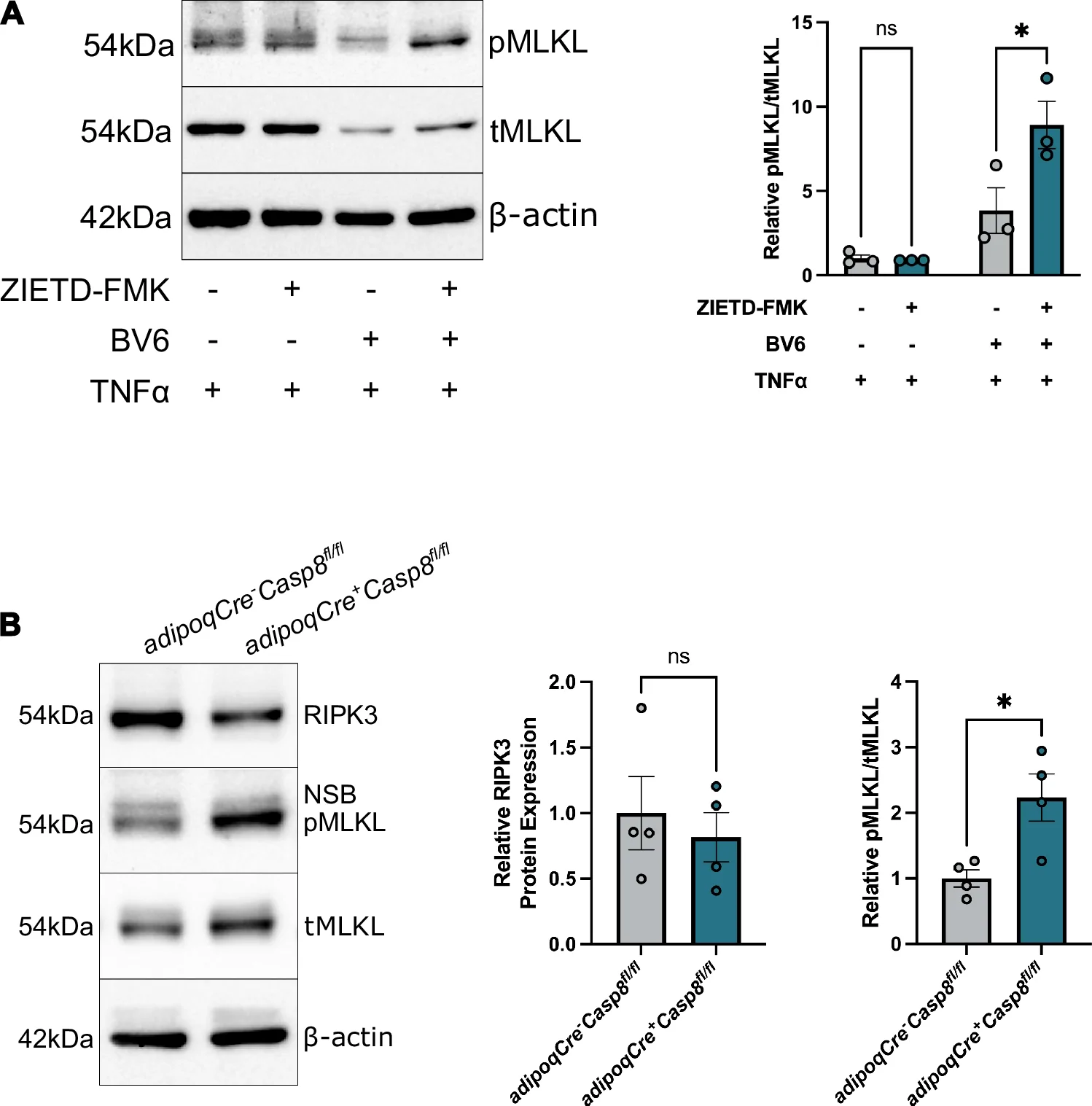
Disruption of caspase-8 activates necroptotic signalling in adipocytes. **A** Immunoblot and quantification of phosphorylated MLKL at S^345^ (pMLKL) and total MLKL (tMLKL) in 3T3-L1 adipocytes treated with or without caspase-8 inhibitor (ZIETD-FMK), with or without cIAP-1/2 inhibitor (BV6), and stimulated with TNFα. β-actin as loading control. *n* = 3 independent experiments. Data are mean ± SEM. * *p* ≤ 0.05 or not significant (ns) by two-way ANOVA. **B** Immunoblot and quantification of RIPK3, phosphorylated MLKL at S^345^ (pMLKL), and total MLKL (tMLKL) protein expression in whole perigonadal WAT from male mice with (*adipoqCre*^*+*^*Casp8*^*fl/fl*^) or without (*adipoqCre*^*-*^*Casp8*^*fl/fl*^) adipocyte-specific knockout of caspase-8 fed up to 20 weeks HFD. β-actin as loading control. *n* = 4 male mice/group. Data are mean ± SEM. * *p* ≤ 0.05 or not significant (ns) by Student’s *t* test. Non-specific binding (NSB). **A**, **B** β-actin shown is representative of multiple blots (See original western blots in Supplementary Information).

Caspase-8 signalling is upregulated in WAT of humans with metabolic dysfunction and a mouse model of diet-induced obesity [30]. Caspase-8 has been demonstrated to suppress necroptosis by cleaving RIPK3 and other mediators of necroptosis in several cell lines [17, 25, 31]. To our knowledge, the regulation of necroptosis by caspase-8 has not been demonstrated in adipocytes. To validate this pathway in adipocytes, we used the 3T3-L1 murine cell line, which is widely used for the study of adipocytes due to the inherent ability to differentiate into cells that accumulate lipid droplets [32, 33]. We and others [34] have found that tumour necrosis factor alpha (TNFα) alone is insufficient to induce cell death in 3T3-L1 adipocytes (Supplementary Fig. S2). Thus, cells were also treated with BV6, a potent antagonist of cellular inhibitors of apoptosis 1 and 2, which facilitate TNFα-dependent caspase-8-mediated apoptosis [35]. Cells were also treated with ZIETD-FMK, a selective caspase-8 inhibitor [36, 37]. Phosphorylation of MLKL was significantly increased with caspase-8 inhibition in vitro (Fig. 2A). To confirm this in vivo, we used previously generated adipocyte-specific caspase-8 knockout mice by adiponectin-Cre (*adipoqCre*) *LoxP* recombination [30]. Consistently, phosphorylation of MLKL was significantly increased in vivo in perigonadal WAT of mice with adipocyte-specific knockout of caspase-8 (*adipoqCre*^*+*^*Casp8*^*fl/fl*^) compared to *adipoqCre*^*-*^*Casp8*^*fl/fl*^ littermates (Fig. 2B) or in mice treated with ZIETD-FMK (Supplementary Fig. S3). Together, these findings demonstrate that caspase-8 negatively regulates necroptotic signalling in adipocytes.

### Disruption of adipocyte caspase-8 reduces adiposity independent of RIPK3

**Fig. 3:**
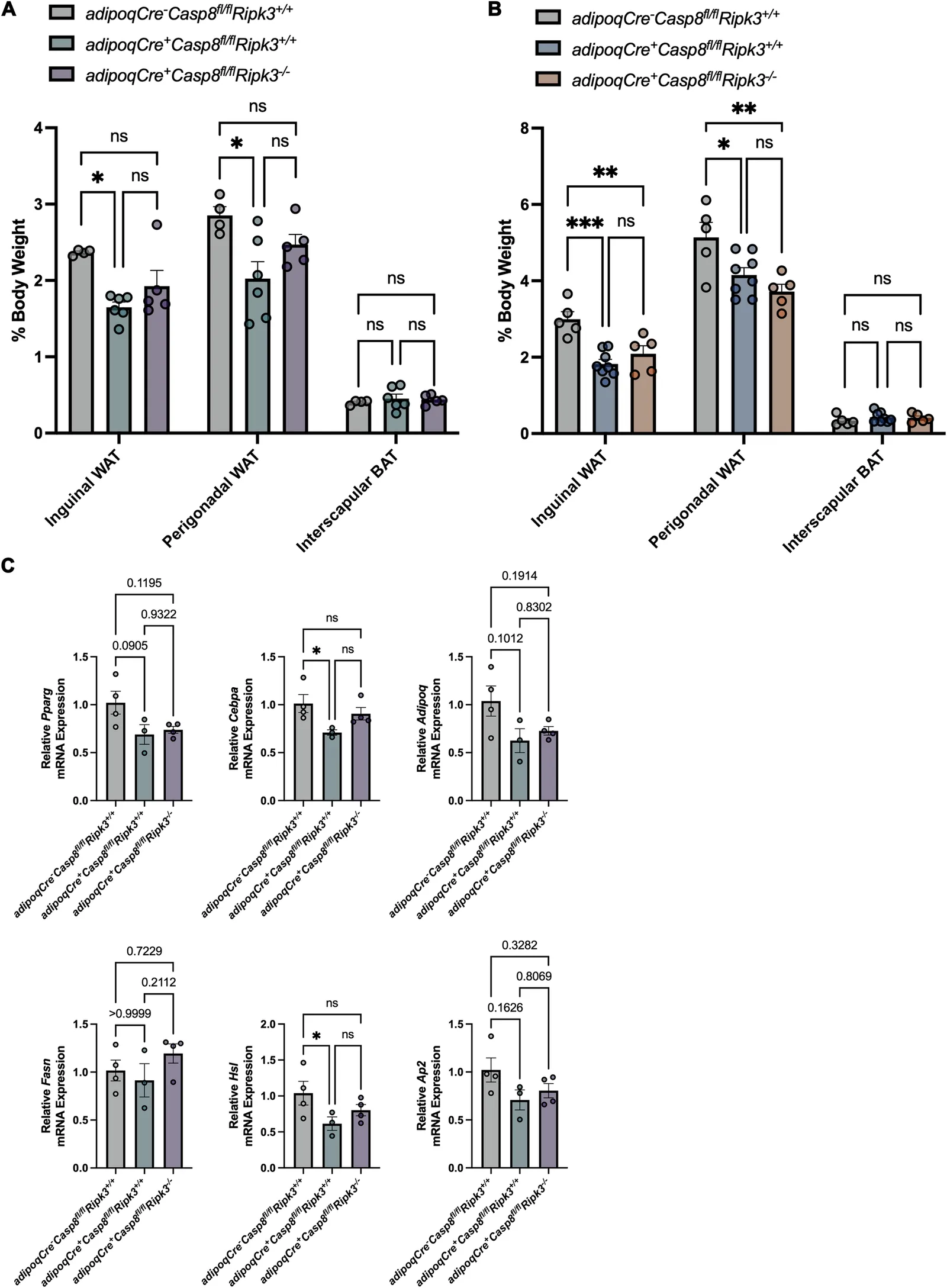
Disruption of adipocyte caspase-8 reduces adiposity independent of RIPK3. Inguinal WAT, perigonadal WAT, and interscapular BAT weights expressed as a percentage of the total body weight of (**A**) male and (**B**) female mice with or without adipocyte-specific *caspase-8* knockout and with or without global deletion of *Ripk3* fed HFD for 16 weeks. **A** *adipoqCre*^*-*^*Casp8*^*fl/fl*^*Ripk3*^*+/+*^ *n* = 4; *adipoqCre*^*+*^*Casp8*^*fl/fl*^*Ripk3*^*+/+*^ *n* = 6; *adipoqCre*^*+*^*Casp8*^*fl/fl*^*Ripk3*^*−/−*^ *n* = 5. **B** *adipoqCre*^*-*^*Casp8*^*fl/fl*^*Ripk3*^*+/+*^ *n* = 5; *adipoqCre*^*+*^*Casp8*^*fl/fl*^*Ripk3*^*+/+*^ *n* = 8; *adipoqCre*^*+*^*Casp8*^*fl/fl*^*Ripk3*^*−/−*^ *n* = 5. **C** Relative mRNA expression of key genes involved in adipogenesis and lipogenesis with or without adipocyte-specific *Casp8* and global *Ripk3* deletion in male mice fed HFD for 16 weeks. *Ywhaz* as endogenous control. *adipoqCre*^*-*^*Casp8*^*fl/fl*^*Ripk3*^*+/+*^ *n* = 4; *adipoqCre*^*+*^*Casp8*^*fl/fl*^*Ripk3*^*+/+*^ *n* = 3; *adipoqCre*^*+*^*Casp8*^*fl/fl*^*Ripk3*^*−/−*^ *n* = 4. Data are mean ± SEM. * *p* ≤ 0.05, ** *p* ≤ 0.01, *** *p* ≤ 0.001, or not significant (ns) by one-way ANOVA with Tukey’s HSD or Kruskal-Wallis test with Dunn’s multiple comparisons test for groups that were not normally distributed.

We previously demonstrated that adipocyte-specific disruption of caspase-8 reduced WAT mass, a phenotype more pronounced in male mice [30]. Given that caspase-8 cleaves RIPK3 to suppress necroptotic signalling, we hypothesized that disruption of adipocyte caspase-8 disinhibited RIPK3 signalling. Thus, we wanted to elucidate the role of RIPK3 in reducing adiposity in mice with adipocyte-specific disruption of caspase-8. To examine this, we generated mice with or without adipocyte-specific disruption of caspase-8 and with or without global loss of *Ripk3*. As before, six-week-old male and female mice were fed HFD for 16 weeks as a model of diet-induced obesity. Consistent with previous findings, adipocyte-specific disruption of caspase-8 reduced WAT mass in both male and female mice (Fig. 3A, B). This was not associated with differences in food intake or locomotion (Supplementary Fig. S4). WAT mass was not further altered with concomitant global deletion of *Ripk3* (Fig. 3A, B). This was consistent with reduced expression of genes involved in adipogenesis and lipogenesis in WAT of male mice with adipocyte-specific disruption of caspase-8 (Fig. 3C). Together, these findings suggest that disruption of adipocyte caspase-8 reduces adiposity independent of RIPK3. As our model utilized global *Ripk3* loss, the specific functions of adipocyte RIPK3 during diet-induced obesity remain to be characterized.

### Generation of adipocyte-specific RIPK3 RHIM knockout mice

**Fig. 4:**
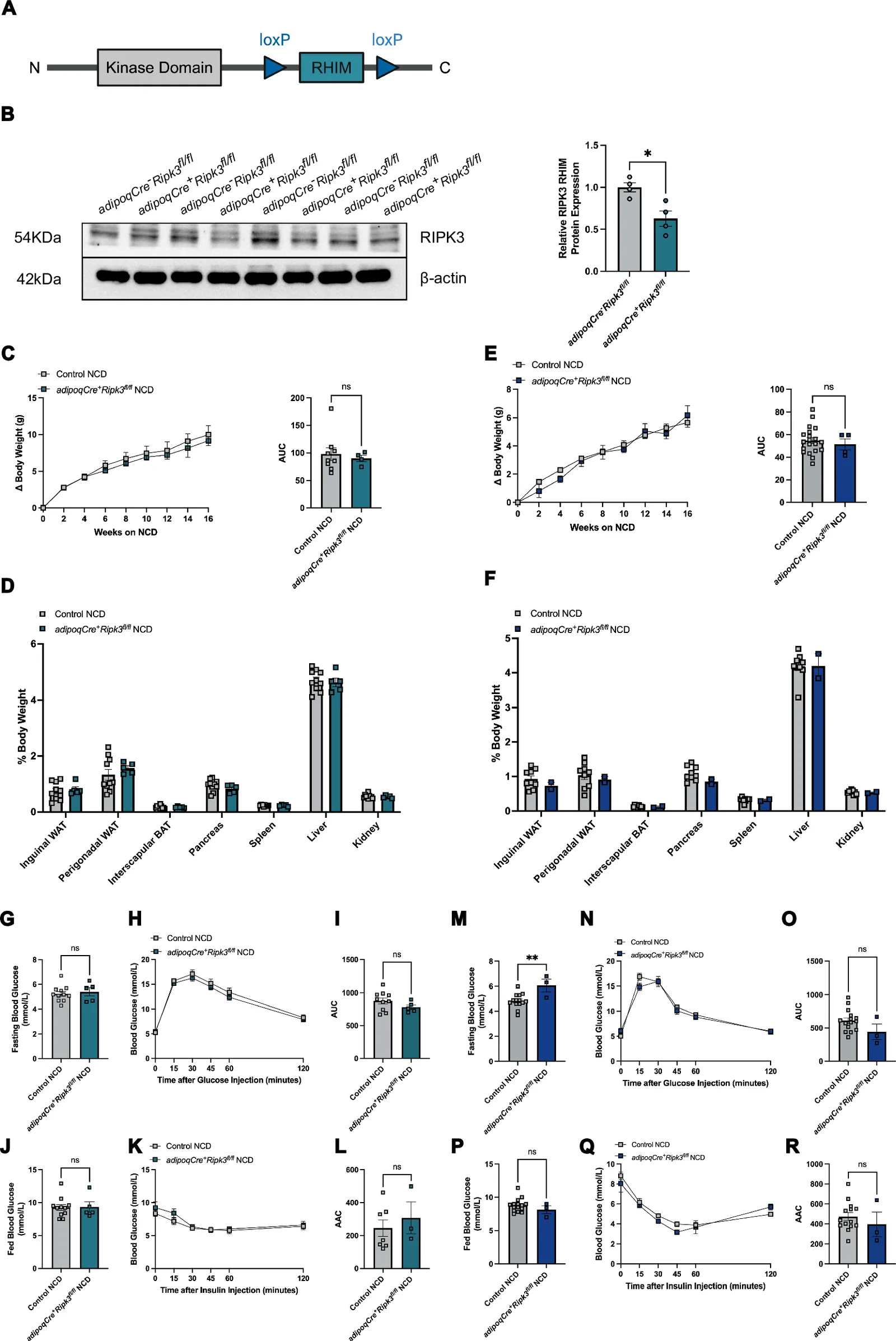
Generation of adipocyte-specific RIPK3 RHIM knockout mice. **A** Schematic representation of the deleted *Ripk3* RIP homotypic interaction motif (RHIM) located at the C-terminus of the *Ripk3* gene in adipocytes using adiponectin-Cre*LoxP* recombination. Created in BioRender. Winer, D. (2026) https://BioRender.com/0o9sj25. **B** Immunoblot (left) and quantification (right) of RIPK3 using an anti-RIPK3 antibody specific to an amino acid sequence near the C-terminus of murine RIPK3 in whole perigonadal WAT from male mice fed HFD for 16 weeks. β-actin as loading control. β-actin shown is representative of multiple blots (See original western blots in Supplementary Information). *n* = 4 male mice/group. Total body weight (g) and area under the curve of (**C**) male and (**E**) female control mice (*adipoqCre*^*-*^*Ripk3*^*+/+*^, *adipoqCre*^*-*^*Ripk3*^*fl/fl*^, *adipoqCre*^*+*^*Ripk3*^*+/+*^: *n* = 9 males; *n* = 20 females) and (**C**) male and (**E**) female adipocyte-specific RIPK3 RHIM knockout mice (*adipoqCre*^*+*^*Ripk3*^*fl/fl*^: *n* = 4 males; *n* = 4 females) fed normal chow diet (NCD) starting at 6–8 weeks of age for 16 weeks. Data are mean ± SEM. Not significant (ns) by Student’s *t* test. Tissue weights expressed as percentage of total body weight of (**D**) male and (**F**) female mice fed NCD for 16 weeks. (Controls: *n* = 10 males; *n* = 9 females; *adipoqCre*^*+*^*Ripk3*^*fl/fl*^: *n* = 5 males; *n* = 2 females). Data are mean ± SEM. Not significant by Student’s *t* test. Fasting blood glucose of (**G**) male and (**M**) female mice fed NCD for 16 weeks. (Controls: *n* = 11 males; *n* = 14 females; *adipoqCre*^*+*^*Ripk3*^*fl/fl*^: *n* = 5 males; *n* = 3 females). Data are mean ± SEM. ** *p* ≤ 0.01 or not significant (ns) by Student’s *t* test. Intraperitoneal glucose tolerance test (1U/kg body weight) and area under the curve (AUC) performed in (**H**, **I**) male and (**N**, **O**) female mice fed NCD for 16 weeks. (Controls: *n* = 10 males; *n* = 15 females; *adipoqCre*^*+*^*Ripk3*^*fl/fl*^: *n* = 5 males; *n* = 3 females). Data are mean ± SEM. Not significant (ns) by Student’s *t* test. Fed blood glucose of (**J**) male and (**P**) female mice fed NCD for 16 weeks. (Controls: *n* = 11 males; *n* = 15 females; *adipoqCre*^*+*^*Ripk3*^*fl/fl*^: *n* = 5 males; *n* = 3 females). Data are mean ± SEM. Not significant (ns) by Student’s *t* test. Intraperitoneal insulin tolerance test (0.5U/kg body weight) and area above the curve (AAC) performed in (**K**, **L**) male and (**Q**, **R**) female mice fed NCD for 16 weeks. (Controls: *n* = 7 males; *n* = 14 females; *adipoqCre*^*+*^*Ripk3*^*fl/fl*^: *n* = 3 males; *n* = 3 females). Data are mean ± SEM. Not significant (ns) by Student’s *t* test.

To further specifically examine the physiological role of adipocyte RIPK3 in diet-induced obesity and metabolic dysfunction, we generated novel mice lacking the C-terminal RHIM of RIPK3 in adipocytes (*adipoqCre*^*+*^*Ripk3*^*fl/fl*^) and littermate controls (*adipoqCre*^*-*^*Ripk3*^*+/+*^, *adipoqCre*^*-*^*Ripk3*^*fl/fl*^, and *adipoqCre*^*+*^*Ripk3*^*+/+*^) using adiponectin-Cre*LoxP* recombination (Fig. 4A), which is highly specific to adipocytes [38]. The RIPK3 RHIM is required for necroptotic induction [39, 40] as it facilitates binding to other RHIM-containing proteins, including receptor-interacting protein kinase 1 (RIPK1). Mouse genotypes were confirmed by conventional PCR. In whole perigonadal WAT, *Cre* mRNA expression was undetected in control mice (Ct < 33, data not shown), and Western blotting using a polyclonal antibody that recognizes the C-terminus of murine RIPK3 detected reduced RIPK3 protein expression in *adipoqCre*^*+*^*Ripk3*^*fl/fl*^ compared to control mice (Fig. 4B), indicating the production of a truncated RIPK3 protein that lacks RHIM [41]. Residual RIPK3 expression may be derived from non-adipocyte cell types, as whole-tissue protein lysates were used. Mice were born at the expected Mendelian ratios (Supplementary Table ST2), indicating that loss of adipocyte RIPK3 RHIM did not result in embryonic lethality. Mice appeared healthy and normal, with no differences in frequency of injury, barbering, or illness across genotypes. Littermate controls (*adipoqCre*^*-*^*Ripk3*^*+/+*^, *adipoqCre*^*-*^*Ripk3*^*fl/fl*^, and *adipoqCre*^*+*^*Ripk3*^*+/+*^) were grouped together for data analysis as there were no observed differences in weight gain or glucose homeostasis in both males and females (Supplementary Fig. S5). Under NCD feeding, no differences in weight gain, body composition, and glucose homeostasis were observed between *adipoqCre*^*+*^*Ripk3*^*fl/fl*^ and littermate controls in both males (Fig. 4C, D, G–L) and females (Fig. 4E, F, M–R).

### Adipocyte RIPK3 RHIM does not contribute to obesity-associated weight gain or adiposity

**Fig. 5:**
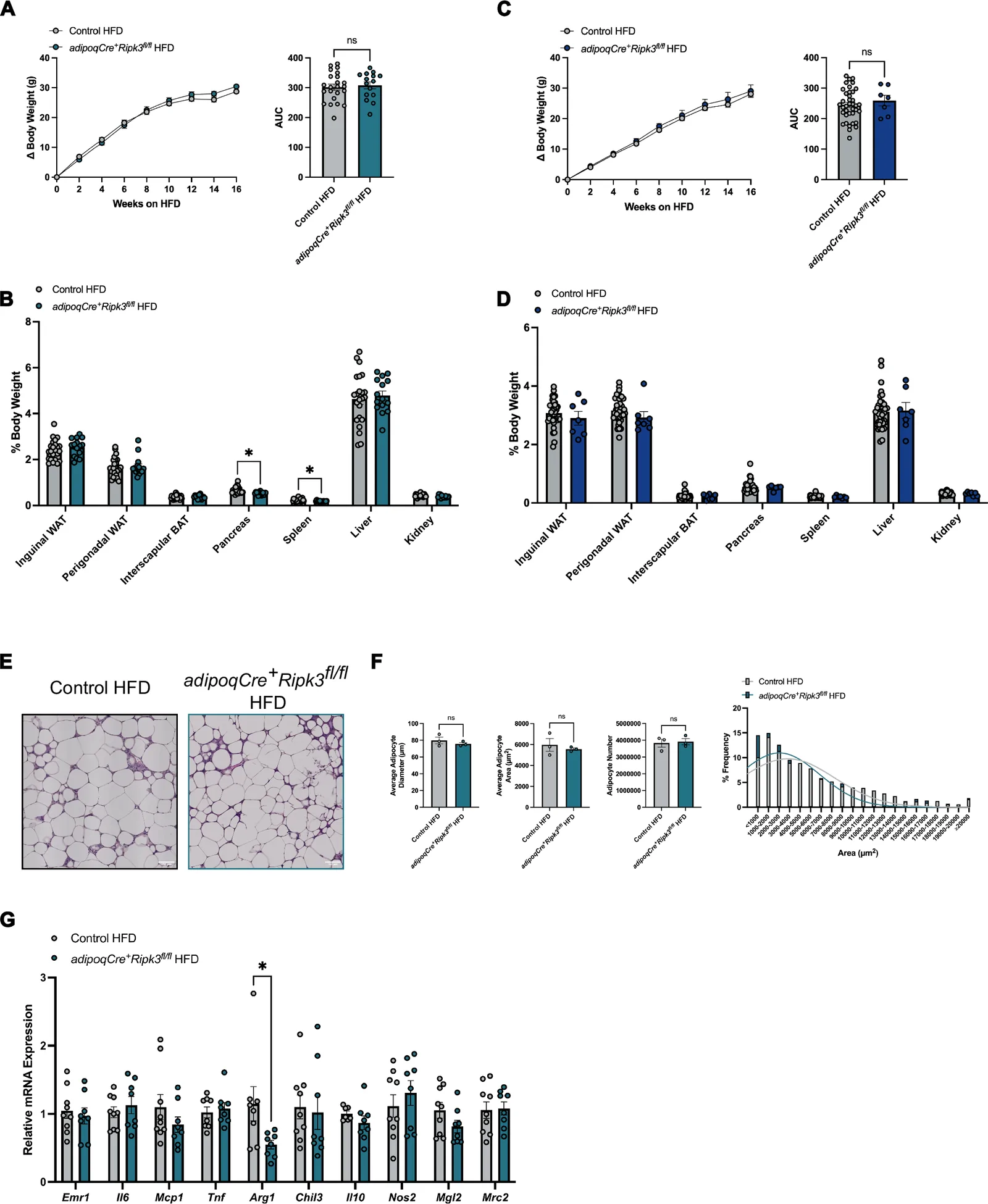
Adipocyte RIPK3 RHIM does not contribute to obesity-associated weight gain or adiposity. Total body weight (g) and area under the curve of (**A**) male and (**C**) female control mice (*adipoqCre*^*-*^*Ripk3*^*+/+*^, *adipoqCre*^*-*^*Ripk3*^*fl/fl*^, *adipoqCre*^*+*^*Ripk3*^*+/+*^: *n* = 23 males; *n* = 38 females) and (**A**) male and (**C**) female adipocyte-specific RIPK3 RHIM knockout mice (*adipoqCre*^*+*^*Ripk3*^*fl/fl*^: *n* = 15 males; *n* = 7 females) fed 60% high-fat diet (HFD) starting at 6–8 weeks of age for 16 weeks. Data are mean ± SEM. Not significant (ns) by Student’s *t* test. Tissue weights expressed as percentage of total body weight of (**B**) male and (**D**) female mice fed HFD for 16 weeks. (Controls: *n* = 23 males; *n* = 38 females; *adipoqCre*^*+*^*Ripk3*^*fl/fl*^: *n* = 15 males; *n* = 7 females). Data are mean ± SEM. * *p* ≤ 0.05 or not significant (ns) by Student’s *t* test. **E** Hematoxylin and eosin-stained whole perigonadal WAT from male mice fed HFD for 16 weeks. Original magnification = 20×. Scale bar = 100 µm. Representative images from *n* = 3 male mice/group. **F** Quantification of (left to right) average adipocyte diameter (µm), average adipocyte area (µm^2^), and estimated number of adipocytes, and (bottom) percent frequency of adipocyte area in whole perigonadal WAT from male mice fed HFD for 16 weeks. *n* = 3 male mice/group. Not significant (ns) by Student’s *t* test. **G** Relative mRNA expression of markers of M1 and M2 macrophages in whole perigonadal WAT from male mice HFD for 16 weeks. *Ywhaz* as endogenous control. (Controls: *n* = 7; *adipoqCre*^*+*^*Ripk3*^*fl/fl*^: *n* = 8). Data are mean ± SEM. * *p* ≤ 0.05 by Student’s *t* test.

To examine the physiological role of adipocyte RIPK3 in weight gain and adiposity, six- to eight-week-old male and female *adipoqCre*^*+*^*Ripk3*^*fl/fl*^ and littermate controls were fed HFD for 16 weeks. HFD-feeding induced significant weight gain in both male and female mice (Fig. 5A, C). There were no differences in weight gain on HFD between *adipoqCre*^*+*^*Ripk3*^*fl/fl*^ and littermate controls (Fig. 5A, C). Perigonadal WAT, inguinal WAT, interscapular brown adipose tissue (BAT), and non-adipose tissue organ weights expressed as a percentage of total body weight were also not different between *adipoqCre*^*+*^*Ripk3*^*fl/fl*^ and littermate controls under HFD-fed conditions (Fig. 5B, D). Notably, male *adipoqCre*^*+*^*Ripk3*^*fl/fl*^ had reduced pancreas and spleen weight compared to male littermate controls on HFD (Fig. 5B). There were no observed differences in morphology, area, or estimated number of adipocytes in perigonadal WAT between male *adipoqCre*^*+*^*Ripk3*^*fl/fl*^ and male littermate controls on HFD (Fig. 5E, F). Similarly, there were no differences in WAT inflammation between male *adipoqCre*^*+*^*Ripk3*^*fl/fl*^ and male littermate controls on HFD, with the exception of reduced *Arg1* mRNA expression (Fig. 5G). Together, these results suggest that the adipocyte RIPK3 RHIM is unlikely to modulate weight gain and adiposity during diet-induced obesity in both male and female mice.

### Adipocyte RIPK3 RHIM does not contribute to obesity-associated loss of glucose homeostasis

**Fig. 6:**
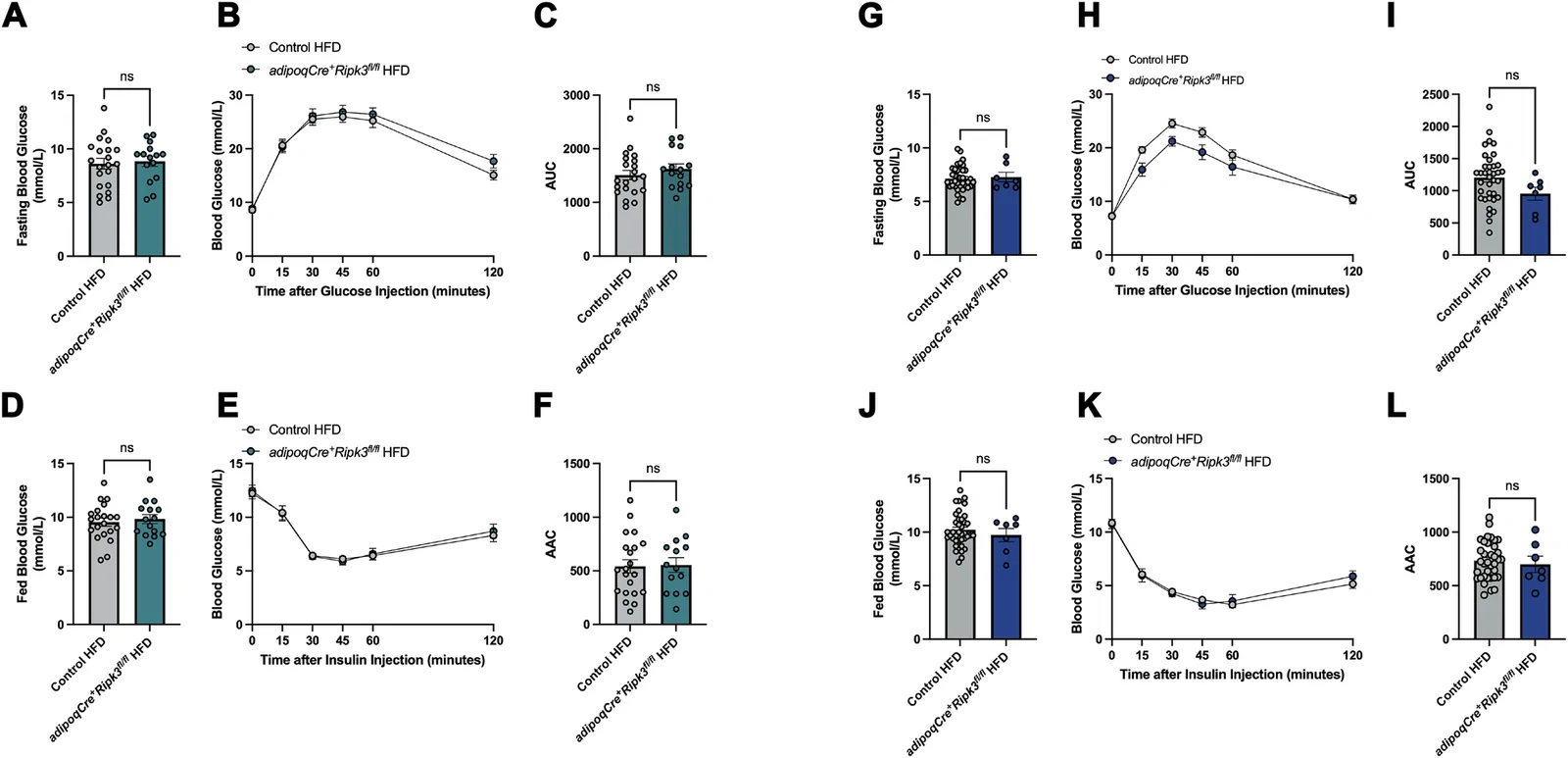
Adipocyte RIPK3 RHIM does not contribute to obesity-associated loss of glucose homeostasis. Fasting blood glucose of (**A**) male and (**G**) female mice fed HFD for 16 weeks. (Controls: *n* = 21 males; *n* = 37 females; *adipoqCre*^*+*^*Ripk3*^*fl/fl*^: *n* = 15 males; *n* = 7 females). Data are mean ± SEM. Not significant (ns) by Student’s *t* test. Intraperitoneal glucose tolerance test (1U/kg body weight) and area under the curve (AUC) performed in (**B**, **C**) male and (**H**, **I**) female mice HFD for 16 weeks. (Controls: *n* = 21 males; *n* = 37 females; *adipoqCre*^*+*^*Ripk3*^*fl/fl*^: *n* = 15 males; *n* = 7 females). Data are mean ± SEM. Not significant (ns) by Student’s *t* test. Fed blood glucose of (**D**) male and (**J**) female mice fed HFD for 16 weeks. (Controls: *n* = 21 males; *n* = 38 females; *adipoqCre*^*+*^*Ripk3*^*fl/fl*^: *n* = 15 males; *n* = 7 females). Data are mean ± SEM. Not significant (ns) by Student’s *t* test. Intraperitoneal insulin tolerance test (1U/kg body weight) and area above the curve (AAC) performed in (**E**, **F**) male and (**K**, **L**) female mice fed HFD for 16 weeks. (Controls: *n* = 21 males; *n* = 36 females; *adipoqCre*^*+*^*Ripk3*^*fl/fl*^: *n* = 14 males; *n* = 7 females). Data are mean ± SEM. Not significant (ns) by Student’s *t* test.

After 12 to 13 weeks of HFD-feeding, both male and female mice developed significant obesity-associated hyperglycemia, characterized by elevated fasting blood glucose and glucose intolerance when undergoing intraperitoneal glucose tolerance testing (Fig. 6A–C, G–I). In both male and female mice, there were no differences in fasting blood glucose (Fig. 6A, G), glucose intolerance (Fig. 6B, C, H, I), fed blood glucose (Fig. 6D, J), or insulin sensitivity demonstrated by intraperitoneal insulin tolerance testing (Fig. 6E, F, K, L) between *adipoqCre*^*+*^*Ripk3*^*fl/fl*^ and littermate controls under HFD-fed conditions. These results suggest that adipocyte RIPK3 RHIM does not contribute to obesity-associated loss of glucose homeostasis.

### Caspase-8 promotes adipogenesis independent of RIPK3

The present findings suggest that adipocyte RIPK3 RHIM does not contribute to obesity or loss of glucose homeostasis in mice. Given that the RIPK3 RHIM facilitates necroptotic induction, the increase in RIPK3 protein expression observed in WAT of mice with diet-induced obesity may indicate unknown RHIM-independent or non-necroptotic functions of RIPK3 in obesity and metabolic dysfunction.

**Fig. 7:**
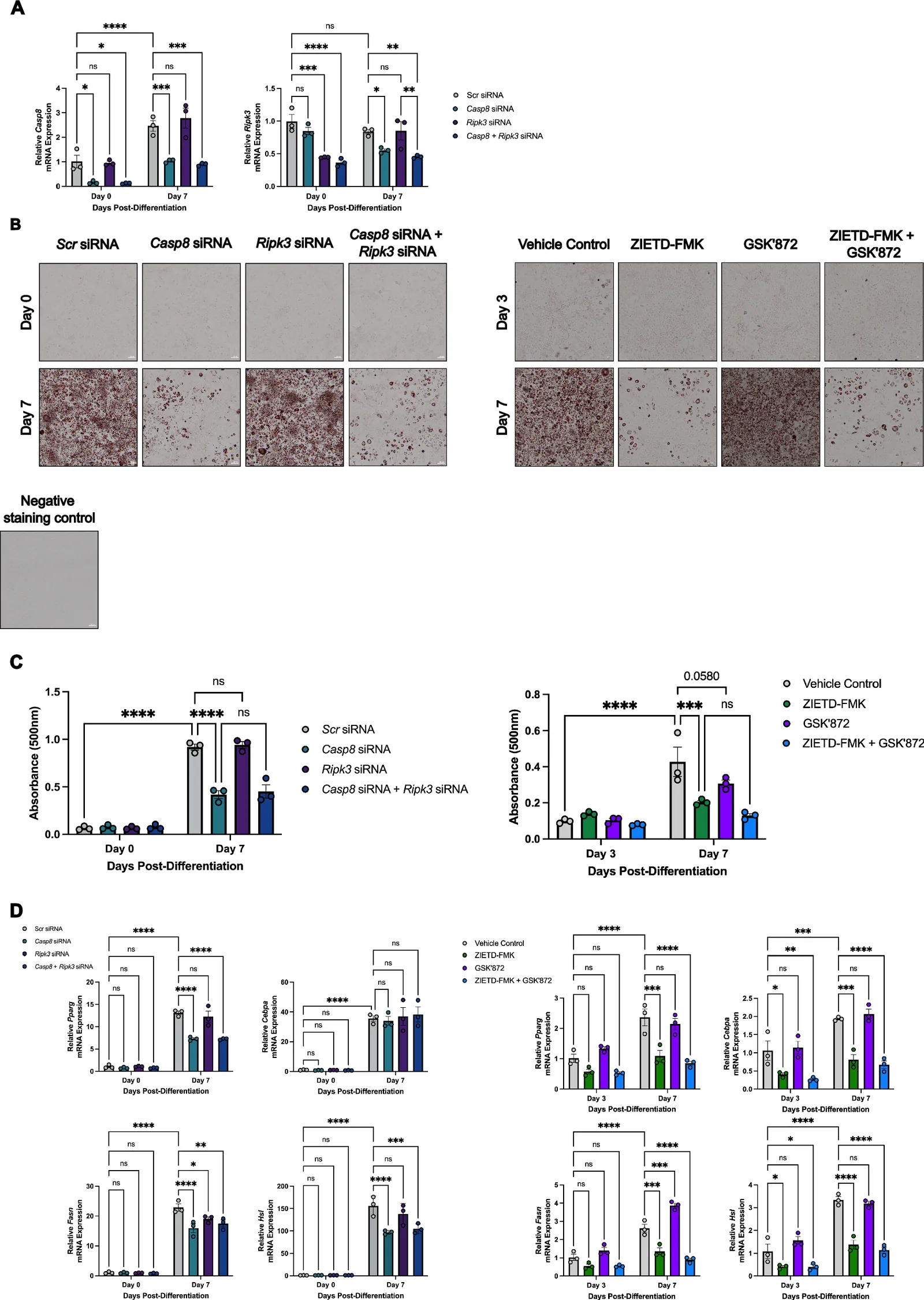
Caspase-8 promotes adipogenesis independent of RIPK3. **A** Relative *Casp8* and *Ripk3* mRNA expression in 3T3-L1 adipocytes with or without *Casp8* and/or *Ripk3* knockdown by siRNA transfection on 0- and 7-days post-transfection. *Bact* as endogenous control. *n* = 3 independent experiments. Data are mean ± SEM. * *p* ≤ 0.05, ** *p* ≤ 0.01, *** *p* ≤ 0.001, **** *p* ≤ 0.0001, or not significant (ns) by two-way ANOVA. **B** Intracellular lipid staining by Oil Red O (ORO) in empty well (negative staining control) or 3T3-L1 adipocytes transfected with (from left to right) *Scr* siRNA, *Casp8* siRNA, *Ripk3* siRNA, *Casp8* and *Ripk3* siRNA (left) on 0 (top row) and 7 days (bottom row) post-differentiation, or treated with media supplemented with (from left to right) vehicle control, 50 µM ZIETD-FMK, 5 µM GSK’872, 50 µM ZIETD-FMK and 5 µM GSK’872 (right), on 3 (top row) and 7 days (bottom row) post-differentiation. Original magnification = 10×. Scale bar = 100 µm. Representative images from *n* = 3 independent experiments. **C** Quantification of ORO staining in 3T3-L1 adipocytes transfected with siRNA (left) on 0- and 7-days post-differentiation or treated with synthetic inhibitors (right) on 3- and 7-days post-differentiation measured by eluting ORO stain with absolute isopropanol and reading absorbance at 500 nm. Data are mean ± SEM. **** *p* ≤ 0.0001 or not significant (ns) by two-way ANOVA. **D** Relative mRNA expression of key markers of mature adipocytes in 3T3-L1 adipocytes with or without *Casp8* and/or *Ripk3* knockdown by siRNA transfection (left) or treated with synthetic inhibitors (right). *Bact* as endogenous control. *n* = 3 independent experiments. Data are mean ± SEM. * *p* ≤ 0.05, ** *p* ≤ 0.01, *** *p* ≤ 0.001, or **** *p* ≤ 0.0001 by two-way ANOVA.

*Casp8*, *Ripk3*, or a combination of both genes were knocked-down in 3T3-L1 pre-adipocytes by siRNA transfection (Fig. 7A). After 48 hours, differentiation was induced. Differentiation and lipid metabolism were assessed by Oil Red O (ORO) staining or qPCR, respectively, at time of differentiation (Day 0) and seven days post-differentiation (Day 7). Differentiation into mature 3T3-L1 adipocytes significantly induced lipid accumulation, as demonstrated by increased ORO staining (Fig. 7B, left). Knockdown of *Casp8* significantly reduced differentiation into mature 3T3-L1 adipocytes; however, this function was demonstrated to be independent of *Ripk3* (Fig. 7B, C, left). Knockdown of *Casp8*, but not *Ripk3*, significantly reduced expression of key mature adipocyte markers: *Pparg*, *Fasn*, and *Hsl*, which was also *Ripk3*-independent (Fig. 7D, left). Similarly, a significant reduction in adipogenesis was also observed when cells were treated with media supplemented with synthetic caspase-8 inhibitor ZIETD-FMK, but not RIPK3 inhibitor GSK’872 (Fig. 7B–D, right). We validated this in vivo using mice fed short- and long-term HFD and injected with 4 mg/kg ZIETD-FMK or vehicle control daily for 5 days. ZIETD-FMK treatment decreased adipogenic gene expression in perigonadal WAT from mice fed short-term HFD, but not long-term HFD, indicating an important role of caspase-8 in WAT expansion (Supplementary Fig. S6). Together, these findings reveal a novel, non-apoptotic role of caspase-8 in regulating adipogenesis, which may be independent of its crosstalk with RIPK3 and necroptotic signalling.

## Discussion

In the present study, we demonstrated that caspase-8 regulates necroptotic signalling in adipocytes. RIPK3 and necroptotic signalling, characterized by phosphorylation of MLKL, was associated with BMI in human WAT and was upregulated in WAT of mice with diet-induced obesity. Decreased adiposity observed in mice with adipocyte-specific loss of caspase-8 was not further altered by concomitant global deletion of RIPK3. To examine the physiological role of RIPK3 specifically in adipocytes, we generated novel mice with adipocyte-specific disruption of RIPK3 RHIM, which is required for induction of necroptosis. However, we demonstrated that adipocyte RIPK3 RHIM is not critical for weight gain, adiposity, or glucose homeostasis. Rather, we identified a novel, non-apoptotic role of caspase-8 in promoting adipogenesis.

Adipocyte death is recognized to contribute to the pro-inflammatory shift in WAT during obesity. Several modes of adipocyte death have been reported in WAT of humans and mouse models of obesity and metabolic dysfunction, including apoptosis [27, 30, 42], as well as necrosis-like modes of cell death including necroptosis [20, 43] and more recently, pyroptosis [6, 44, 45]. It is plausible that several modes of adipocyte death occur simultaneously in this context. Identifying the mode of adipocyte death that most substantially drives WAT inflammation during obesity may lend insight into targeting these pathways as a therapeutic strategy.

Our group previously reported that caspase-8 signalling, a central regulator of apoptosis, is increased in WAT of humans and mouse models of obesity and metabolic dysfunction [30]. We reported that adipocyte-specific disruption of caspase-8 reduced adiposity and improved metabolic dysfunction in obese mice [30]. Complementary to our findings, complete ablation of adipocytes by caspase-8-mediated apoptosis using the fat apoptosis through targeted activation of caspase-8 (FAT-ATTAC) mouse model [46] induces macrophage infiltration in WAT [8]. Macrophages were predominantly of an alternatively activated phenotype (“M2”) [8], rather than the classically activated, pro-inflammatory phenotype (“M1”) that accompanies WAT inflammation and metabolic dysfunction during obesity [7]. This brings into question whether other modes of cell death, such as necrosis-like adipocyte death–characterized by loss of membrane integrity and leakage of intracellular contents [26]–could trigger pro-inflammatory macrophage infiltration in WAT during obesity [8].

The role of necroptosis in driving systemic inflammation is underscored by studies in mice with global deletion of necroptotic suppressors, including caspase-8 and fas-associated death domain-containing protein (FADD). Global deletion of caspase-8 [47, 48] or FADD [49, 50] triggers excessive necroptosis and inflammation, leading to embryonic lethality. Consistent with this, we also demonstrated that caspase-8 regulates adipocyte necroptosis. To inhibit caspase-8, we used ZIETD-FMK, a selective caspase-8 inhibitor that binds to the catalytic active site of caspase-8, blocking its activity [36, 37]. Our results are consistent with work demonstrating that caspase-8 cleaves RIPK3 [51] via its catalytic active site [52], thereby blocking phosphorylation of MLKL and necroptosis.

In the context of obesity and metabolic dysfunction, investigation of necroptosis has largely been represented by studies of obese mice with global deletion or inhibition of key necroptotic mediators: RIPK1, RIPK3, and MLKL. Thus, our data are strengthened by the use of mice with adipocyte-specific deletion of RIPK3 RHIM to study the role of adipocyte necroptosis in obesity. In canonical necroptosis, RHIM-mediated complex formation between RIPK1 and RIPK3 induces phosphorylation of MLKL for the execution of necroptosis [39]. Bone marrow-derived dendritic cells and embryonic fibroblasts isolated from mice with global deletion of RIPK3 RHIM were protected from lipopolysaccharide and TNFα-induced necroptosis, respectively, demonstrating that the RHIM is essential for RIPK3-mediated necroptosis [41].

We demonstrated that inactivation of the RIPK3 RHIM in adipocytes does not alter adiposity or glucose homeostasis in mice with diet-induced obesity. This is in line with studies demonstrating that global deletion of necroptotic effector MLKL in obese mice only modestly reduced WAT inflammation [53] and did not influence hepatocyte death or hepatic inflammation [20]. Similarly, while RIPK1 kinase activity is essential for necroptosis [54], kinase inactive RIPK1 did not alter weight gain, adiposity, or metabolic dysfunction in obese mice [55]. This minimal contribution of necroptosis in obesity is further highlighted by studies demonstrating improvement of obesity-associated metabolic dysfunction in mice with adipocyte-specific deletion of key necroptotic suppressors, such as caspase-8 [30] and FADD [56]. Specifically, it is intriguing that loss of these suppressors does not induce WAT inflammation by disinhibition of adipocyte necroptosis. Interestingly, necroptosis was suggested to dampen the pro-inflammatory response induced by TNFα and lipopolysaccharide stimulation by transcriptional repression of pro-inflammatory cytokine production through rapid cell death [57–59]. Collectively, these results suggest that the inflammatory outcomes of necroptosis are cell-specific and may not be as inflammatory relative to other modes of adipocyte death. As diet-induced obesity induces a large influx of immune cells into WAT, further investigation may be warranted to study the role of necroptosis in these WAT immune cell subsets.

It is also striking that we observed an upregulation of RIPK3 and phosphorylation of MLKL in WAT of mice with diet-induced obesity in the presence of strong necroptotic suppressors. Further work is needed to clarify if this upregulation indicates increased necroptotic activation or serves other functions in adipocytes [21]. RIPK3 has been demonstrated to have cell-specific, non-necroptotic functions, including immune modulation [41, 60] and regulation of apoptosis, through crosstalk with caspase-8. Recently, RIPK3-caspase-8 signalling in myeloid cells was demonstrated to promote WAT inflammation in obese mice [53]. Furthermore, global deletion of RIPK3 exacerbated WAT inflammation and metabolic dysfunction in obese mice [27, 28] by sensitizing mice to caspase-8-mediated adipocyte apoptosis [27]. While apoptosis is not known to elicit an immune response, caspase-8 is increasingly reported to exhibit functions beyond cell death [61–63]. Together, these findings suggest that RIPK3 may act through mechanisms that do not involve necroptosis in adipocytes, including limiting deleterious effects mediated by caspase-8 signalling and adipocyte apoptosis. Specifically, we identified a novel, non-apoptotic function of caspase-8 in promoting adipogenesis, independent of adipocyte RIPK3. Adipocyte hypertrophy, in the absence of obesity, was demonstrated to induce adipocyte death and macrophage infiltration [6]. Thus, this finding supports the notion that caspase-8 may drive adipocyte hypertrophy during obesity, potentially leading to WAT expansion, adipocyte apoptosis, and WAT inflammation.

Peroxisome proliferator-activated receptor gamma (PPARγ) is a master regulator of lipid uptake and adipogenesis, such that inactive PPARγ impairs normal adipocyte function. Mature 3T3-L1 adipocytes stimulated with TNFα only [64] or in conjunction with cycloheximide [64, 65] were reported to induce caspase cascade-mediated PPARγ cleavage, preventing its nuclear localization and rendering it inactive, thereby impairing normal adipocyte function. In parallel, our present findings build on the existing literature by demonstrating the regulatory role of caspase-8 in adipogenesis under basal conditions, in the absence of inflammatory stimuli and adipocyte death. In the present study, we disrupted caspase-8 function in 3T3-L1 adipocytes either by siRNA knockdown of *Casp8* during the pre-adipocyte stage to ensure maximal knockdown occurred at the time of differentiation (72 h post-transfection) or by differentiation media supplemented with pharmacological inhibitor ZIETD-FMK, whereas previous work used pharmacological inhibitors in fully mature adipocytes. Our results suggest that caspase-8 is required for adipocyte differentiation, indicating a non-apoptotic, regulatory role in adipogenesis.

Limitations should be considered during the interpretation of findings from this study. This work targets RIPK3 as an essential but not exclusive player in necroptosis. Additionally, the production of truncated RIPK3 protein does not limit the action of the RIPK3 kinase domain. Therefore, further work is needed to investigate the role of the RIPK3 kinase domain during obesity, which has been demonstrated to limit apoptosis [29, 66].

Collectively, we observed an upregulation of necroptotic signalling in both human and murine WAT during obesity. We demonstrated that the RIPK3 RHIM in adipocytes is not critical to obesity and glucose homeostasis. Further work is needed to identify non-necroptotic functions of RIPK3 in WAT during obesity. Beyond its essential role in several cell death pathways, we also identified a novel, non-apoptotic role of caspase-8 in regulating adipogenesis. Understanding the adipocyte-specific functions of these cell death mediators will offer insight into the molecular mechanisms underlying obesity and metabolic dysfunction.

## Materials and methods

### Mice

All mice were housed in a specific pathogen-free facility maintained at 21 °C, 30–50% humidity, and 12-hour light/12-hour dark cycle, and fed normal irradiated chow diet *ad libitum* (6% fat, 18% protein, 44% carbohydrates; #2918; Teklad Global; Lafayette, Indiana, USA). Starting at 6 weeks of age, C57BL/6 J (strain #000664; The Jackson Laboratory; Bar Harbor, Maine, USA) were randomly assigned to be fed normal irradiated chow diet (#2918; Teklad Global) or HFD (60% fat, 16% protein, 24% carbohydrates; #F3282; Bio-Serv; Flemington, New Jersey, USA) for 12 weeks. No blinding was done. Adipocyte-specific caspase-8 knockout mice were generated and genotyped as previously described [30, 67]. Adipocyte-specific caspase-8 knockout mice were also crossed with mice with global deletion of RIPK3 (*Ripk3*^−/−^) on a C57BL/6 J background gifted by Dr. Anne Hakem and Dr. Razqallah Hakem to generate heterozygous offspring (*adipoqCre*^-^*Casp8*^*fl/+*^*Ripk3*^+/−^ and *adipoqCre*^+^*Casp8*^*fl/+*^*Ripk3*^+/−^). These mice were then crossed to generate littermate *adipoqCre*^-^*Casp8*^*fl/fl*^*Ripk3*^*+/-*^ and *adipoqCre*^+^*Casp8*^*fl/fl*^*Ripk3*^*+/−*^ mice, which were further crossed to generate littermate *adipoqCre*^*-*^*Casp8*^*fl/fl*^*Ripk3*^+/+^, *adipoqCre*^*+*^*Casp8*^*fl/fl*^*Ripk3*^+/+^, and *adipoqCre*^*+*^*Casp8*^*fl/fl*^*Ripk3*^−/−^ mice. Specifically, this final crossing generated control mice (*adipoqCre*^*-*^*Casp8*^*fl/fl*^*Ripk3*^*+/+*^), mice with adipocyte-specific deletion of caspase-8 (*adipoqCre*^*+*^*Casp8*^*fl/fl*^*Ripk3*^+/+^), and mice with adipocyte-specific deletion of caspase-8 and global deletion of RIPK3 (*adipoqCre*^*+*^*Casp8*^*fl/fl*^*Ripk3*^−/−^).

To generate adipocyte-specific RIPK3 RHIM knockout mice, *adipoqCre*^+^ mice (strain #010803; The Jackson Laboratory) [68] were crossed with *Ripk3*^*fl/+*^ mice (strain #030284; The Jackson Laboratory) [41] to generate *adipoqCre*^*+*^*Ripk3*^*fl/+*^ and *adipoqCre*^*-*^*Ripk3*^*fl/+*^ mice. These mice were intercrossed to generate adipocyte-specific RIPK3 RHIM knockout mice (*adipoqCre*^*+*^*Ripk3*^*fl/fl*^) and littermate controls, which were maintained on a C57BL/6 background.

Mouse genotypes were assessed by conventional PCR of DNA extracted from ear notches obtained from 12- to 14-day-old pups. Genotyping primers for *adipoqCre* were used as previously described [69]. Primer sequences 5’-CGCTTTAGAAGCCTTCAGGTTGAC-3’, 5’-GCAGGCTCTGGTGACAAGATTCATGG-3’, and 5’-CCAGAGGCCACTTGTGTAGCG-3’ were used for *Ripk3*^−/−^ genotyping. The Jackson Laboratory genotyping primers #12240, #33351, and #33352 were used for *Ripk3*^*fl/fl*^ genotyping. The same PCR protocol for *adipoqCre* was used for *Ripk3*. PCR protocol was used as previously described [69].

Starting at 6–8 weeks of age, mice were randomly assigned to be fed a normal irradiated chow diet (#2918; Teklad Global) or HFD (60% fat, 16% protein, 24% carbohydrates; #F3282; Bio-Serv) for the duration indicated. Sample size was determined based on previous studies [30, 69]. Male and female mice were used as indicated. Mice were excluded from the study if injured, barbering, or ill, which did not occur at a different frequency between experimental groups. All animal experimental procedures were in accordance with the Canadian Council on Animal Care guidelines and approved by the St. Michael’s Hospital Animal Care Committee (protocol #948, #960).

### In vivo metabolic studies

After 12 weeks of NCD or HFD-feeding, glucose tolerance tests were performed in mice fasted overnight (16 h) using 1 g of glucose per kilogram of body weight glucose solution injected peritoneally. After 13 weeks of NCD or HFD-feeding, glucose tolerance tests were performed in mice fasted 4 h using 0.5- or 1-units, respectively, of insulin lispro (100 units per millilitre Humalog^®^; Eli Lilly; Indianapolis, Indiana, USA) per kilogram of body weight injected peritoneally. Blood glucose was measured by tail vein sampling at 0-, 15-, 30-, 45-, 60-, and 120 min after injection using a Contour® Next glucometer (Ascensia Diabetes Care; Parsippany, New Jersey, USA). Area under the curve (glucose tolerance test) and area below the curve (insulin tolerance test) were approximated by the trapezoidal rule. Mice were excluded from data analysis if technical error occurred during glucose or insulin injection. Metabolic caging studies were performed as previously described [30].

### In vivo caspase-8 inhibition

Control mice fed HFD for 2 or 7–9 weeks were treated with DMSO vehicle control or 4 mg/kg caspase-8 inhibitor (ZIETD-FMK; #HY-101297; MedChem Express; Monmouth Junction, New Jersey, USA) dissolved in PBS by intraperitoneal injection daily for a total of 5 days. Perigonadal WAT was harvested and processed for qPCR as described below.

### Histology

Mouse perigonadal WAT was dissected and fixed in 10% buffered formalin. After a minimum of 48 hours, tissues were processed using the TP1020 Tissue Processor (Leica; Wetzlar, Germany) and embedded in paraffin using the HistoCore Arcadia Embedding Center (Leica). Perigonadal WAT was cut at 5 µm thickness using the RM2145 Rotary Microtome (Leica) and stained with hematoxylin and eosin using the ST5010 Autostainer XL (Leica). Whole slides images were taken using the AxioScan.Z1 slide scanner (Zeiss; Oberkochen, Germany). Five representative 1000 µm × 1000 µm sections were taken from each whole slide image for analysis. Adipocytes size was estimated using the Adiposoft plug-in [70] for FIJI [71] with manual correction for adipocytes that were incorrectly labelled. At least 400 adipocytes were counted for each sample. Adipocyte number was estimated using adipocyte diameter and adipose tissue mass, as previously described [72].

### RNA extraction and real-time PCR (qPCR)

100 mg adipose tissue or 3T3-L1 adipocytes were lysed using 1 mL TRIzol reagent (#15596026; Invitrogen; Waltham, Massachusetts, USA). RNA was reverse transcribed using the M-MLV reverse transcriptase and random primers (#4368814; Applied Biosystems™; Waltham, Massachusetts, USA). Real-time PCR (qPCR) was performed using SYBR Green (GB-Amp™ PowerGreen™ InFluor™ Green qPCR Mix (Low ROX); #P2101-02ax; GeneBio Systems, Inc.; Burlington, Ontario, Canada) and primer sequences listed in Supplementary Table ST3. Reactions were performed in the QuantStudio 7 qPCR System (#4485701; Applied Biosystems™). Data analysis was performed using the comparative Ct method (ΔΔCT).

### Protein extraction and Western blotting

Complete radioimmunoprecipitation assay (cRIPA) buffer (#sc-24948A; Santa Cruz Biotechnology; Dallas, Texas, USA) was added to adipose tissue. For human samples, adipose tissue collected from around the pancreas and omental adipose tissue collected during bariatric surgery were used. Aggregated demographic information of adipose tissue donors is listed in Supplementary Table ST1. For murine samples, perigonadal and inguinal adipose tissue depots were used. Samples were homogenized (#9002755; Qiagen; Germany) then sonicated briefly on ice. Lysates were centrifuged at 12000 × *g* for 15 min, 4 °C to remove the fat cake. Supernatant was transferred to new tubes, and protein was quantified by Bradford protein assay (#BRA222; BioShop Canada; Burlington, Ontario, Canada). After protein quantification, 2X Laemmli sample buffer (#1610737; Bio-Rad; Hercules, California, USA) was added to 40 µg of protein and boiled at 95°C for 5 minutes. Samples were separated using a 10% sodium dodecyl sulfate-polyacrylamide gel electrophoresis (SDS-PAGE) gel made in-house in Tris-Glycine-SDS running buffer (25 mM Tris, 192 mM glycine, and 0.1% SDS; #811-570-FL; Wisent Inc.; Saint-Jean-Baptiste, Québec, Canada) alongside a protein standard (#1610373; Bio-Rad). Proteins were transferred (#1704150EDU; Bio-Rad) in transfer buffer (#10026938, Bio-Rad) to a 0.2 µm nitrocellulose membrane (#1620112; Bio-Rad). The membrane was briefly rinsed in Tris-buffered saline (20 mM Tris, 150 mM NaCl, pH 7.6) with 0.1% Tween-20 (#TWN510.500; BioShop Canada) (TBST), then blocked in 5% skim milk TBST for 1 h at room temperature on an orbital shaker. After blocking, the membrane was washed three times for 5 min each with TBST. Membranes were then incubated overnight at 4 °C in primary antibody diluted in 5% bovine serum albumin (#ALB001.100; BioShop Canada) TBST. The following primary antibodies were used: RIPK3 (1:1000; #NBP1-77299; Novus Biologicals; Centennial, Colorado, USA), phospho-MLKL S^358^ (human reactivity: 1:1000; #ab187091; Abcam; Cambridge, United Kingdom), phospho-MLKL S^345^ (mouse reactivity: 1:1000; #37333; Cell Signalling Technology; Danvers, Massachusetts, USA), total-MLKL (human reactivity: 1:1000; #ab184718; Abcam; mouse reactivity: 1:1000; #26539; Cell Signalling Technology), or β-actin (1:10 000; #A2228; Sigma-Aldrich; St. Louis, Missouri, USA). The following day, membranes were briefly rinsed with tap water to remove unbound antibody, then washed three times for 5 minutes each with TBST. After washing, membranes were incubated with horseradish peroxidase (HRP)-conjugated secondary antibody diluted in 5% skim milk TBST for 1 h at room temperature. The following secondary antibodies were used: HRP-conjugated polyclonal goat anti-mouse antibody (1:2000 for MLKL; 1:5000 all others; #AB6789; Abcam) or HRP-conjugated polyclonal goat anti-rabbit antibody (1:2000 for phospho-MLKL; 1:5000 for all others; #AB6721; Abcam). As with the primary antibody, membranes were then briefly rinsed with tap water and washed three times for 5 min each with TBST. Membranes were incubated in enhanced chemiluminescence reagent (#45002401; Cytiva; Marlborough, Massachusetts, USA) for phospho-MLKL and (#32106; Thermo Fisher Scientific; Waltham, Massachusetts, USA) for all others and exposed using the ChemiDoc™ Touch Imaging System (Bio-Rad). The membrane used to detect phospho-MLKL was stripped (Restore PLUS Western Blot Stripping Buffer; #46430; Thermo Fisher Scientific) for 10 min with vigorous shaking. The membrane was then blocked and re-incubated in primary total-MLKL antibody and the appropriate secondary antibody as described. Densitometric analysis was performed on Image Lab software (Bio-Rad). Full unedited Western blots are available in Supplementary Information.

### 3T3-L1 adipocyte culture

3T3-L1 pre-adipocytes (#SP-L1-F; Zen-Bio; Durham, North Carolina, USA) were seeded in 6-well plates (#83.3920300; Sarstedt; Nümbrecht, Germany). Cells were expanded in complete growth medium containing Dulbecco’s Modified Eagle’s Medium (DMEM; #319-015-CL; Wisent Inc.) supplemented with 10% fetal bovine serum (FBS; #080-150-EL; Wisent Inc.), 1 mM sodium pyruvate (#600-110-EL; Wisent Inc.), and 1% penicillin-streptomycin solution (#450-201-EL; Wisent Inc.).

Differentiation into mature adipocytes was induced by differentiation media composed of complete growth medium supplemented with 1 µM dexamethasone (#D2915; Sigma-Aldrich), 500 µM IBMX (#I7018; Sigma-Aldrich), and 10 µg/mL insulin (#I9728; Sigma-Aldrich) for 3 days. Mature adipocyte cultures were cultured in maintenance media composed of complete growth media supplemented with 10 µg/mL insulin (#I9728; Sigma-Aldrich) and refreshed every 2 days.

To study the role of caspase-8 in adipocyte necroptosis, 9 days post-differentiation, cells were washed twice with sterile PBS, then serum-starved for 4 h with serum-free growth media. After serum starvation, cells were washed with sterile PBS and treated with DMSO (#DMS666; BioShop Canada), DMSO and 10 µM cellular inhibitor of apoptosis and X-linked inhibitor of apoptosis antagonist (BV6; #HY-16701; MedChem Express), 50 µM caspase-8 inhibitor (ZIETD-FMK; #FMK007; R&D Systems; Minneapolis, Minnesota, USA), or 50 µM ZIETD-FMK and 10 µM BV6. After 1 h, cells were stimulated with 20 ng/mL TNFα (#315-01 A; PeproTech; Cranbury, New Jersey, USA) to induce cell death. After a total of 24 h from treatment, cells were washed twice with PBS, then scraped in the appropriate lysis buffer for RNA or protein extraction, as described.

To study the role of caspase-8 and RIPK3 in adipogenesis, differentiation was induced with differentiation media containing DMSO vehicle control, 50 µM caspase-8 inhibitor (ZIETD-FMK; #HY-101297; MedChem Express), 5 µM RIPK3 inhibitor (GSK-872; #HY-101872; MedChem Express), or both 50 µM caspase-8 inhibitor and 5 µM RIPK3 inhibitor for 3 days. After 3 days, cells were washed with ice-cold PBS and lysed for RNA extraction (Day 3 timepoint). Media was replaced with maintenance media containing the same inhibitors and refreshed every 2 days. After 7 days post-differentiation, cell culture media were collected, and cells were washed and lysed for RNA extraction (Day 7 timepoint) as described.

### siRNA knockdown in 3T3-L1 adipocyte culture

3T3-L1 cells were seeded at 1.65–2 × 10^5^ cells/well in a 6-well plate (#83.3920300; Sarstedt; Germany) with complete growth media. After 24 , cells were washed twice with warm phosphate-buffered saline (PBS). Cells were transfected with 10 nM *Scr* siRNA (#4390843; Thermo Fisher Scientific), 10 nM *Casp8* siRNA (#4390771, s63393; Thermo Fisher Scientific), 10 nM *Ripk3* siRNA (#4390771, s80756; Thermo Fisher Scientific), or 10 nM *Casp8* siRNA and 10 nM *Ripk3* siRNA-lipofectamine (#13778100; Thermo Fisher Scientific) complexes diluted in optiMEM (#31985062; Thermo Fisher Scientific) and allowed to incubate for 8 hours in anti-biotic-free growth media. The difference in volume of total siRNA added per well was adjusted with 10 nM *Scr* siRNA for *Scr* siRNA and single-knockdown groups. After 8 h, media was replaced with complete growth media. Cells were left to proliferate to 100% confluence for two days. Cells were washed with ice-cold PBS and lysed for RNA extraction (Day 0 timepoint), or differentiation was induced with differentiation media (Day 7 timepoint). After 3 days, media was replaced with maintenance media and refreshed every 2 days. After 7 days post-differentiation, cell culture media were collected, and cells were washed and lysed for RNA extraction as described.

### Oil red O staining and imaging

Oil Red O stock solution was made with 0.7 grams Oil Red O powder (#O-0625; Sigma-Aldrich), 200 millilitres of absolute isopropanol, stirred overnight, filtered through a 0.2 µm strainer, and stored at 4 °C for up to a year. Oil Red O working solution was made 6 parts Oil Red O stock solution to 4 parts Milli-Q water. Cells were washed twice in PBS. Cells were fixed in 4% paraformaldehyde diluted in PBS (#28906; Thermo Fisher Scientific) for 5 minutes followed by fresh fixative for 1 h at room temperature. Cells were washed with Milli-Q water to remove excess fixative then washed in 60% isopropanol for 5 min.

Subsequently, Oil Red O working solution was added for 10 min. The stain was removed cells were washed thoroughly with tap water. 16 microscope images for each experimental group were taken on the BioTek Cytation 5 Cell Imaging Multimode Reader (Agilent; Santa Clara, California, USA). Cells were incubated in absolute isopropanol for 10 m with gentle shaking. 250 µL of the eluted stain was measured in duplicate at 500 nm using the BioTek Synergy Neo Multimode Plate Reader (Agilent).

### Statistics

Data are presented as mean ± standard error of the mean (SEM). Data normality was assessed by Shapiro-Wilk test. Unless otherwise indicated in figure legends, data was analyzed by two-tailed unpaired Student’s *t* test for comparisons between two groups if data was normally distributed, two-tailed unpaired Mann-Whitney *U*-test for comparisons between two groups if data was not normally distributed, one-way ANOVA with Tukey’s honestly significant difference (HSD) between more than two groups, two-way ANOVA with Tukey’s HSD for multiple comparisons between more than two groups across multiple independent variables, or simple linear regression to assess the relationship between two continuous variables using GraphPad Prism version 10.4.0 for MacOS (GraphPad Software; Boston, Massachusetts, USA). Statistical significance was defined as *p* value ≤ 0.05: Not significant (ns) denotes *p* > 0.05, * denotes *p* ≤ 0.05, ** denotes *p* ≤ 0.01, *** denotes *p* ≤ 0.001, or **** denotes *p* ≤ 0.0001.

### Ethics

Human adipose tissue was dissected from the omental fat attached to donor pancreases diverted to research (approved by Trillium Gift of Life Network, Toronto, Ontario) provided by Dr. Herbert Y. Gaisano and approved by Research Ethics Board at the University Health Network and University of Toronto. Informed consent was obtained from all subjects. The study was performed in accordance with the Declaration of Helsinki. Aggregated demographic information of adipose tissue donors are listed in Supplementary Table ST1.

## Supplementary information

Supplementary Figure S1

Supplementary Figure S2

Supplementary Figure S3

Supplementary Figure S4

Supplementary Figure S5

Supplementary Figure S6

Supplementary Table ST1

Supplementary Table ST2

Supplementary Table ST3

Full, Unedited Western Blots

## Data Availability

All data generated or analysed during this study are included in this published article and its supplementary information files.
