# Supplementary material for: Adipocyte caspase-8 but not RIPK3 promotes adiposity
Source: Cell Death Discov. 2026 Jun 23;12:356. doi: 10.1038/s41420-026-03201-z (PMC13547226; doi:10.1038/s41420-026-03201-z)
Supplement: Supplementary file 1 [file 41420_2026_3201_MOESM10_ESM.pdf]

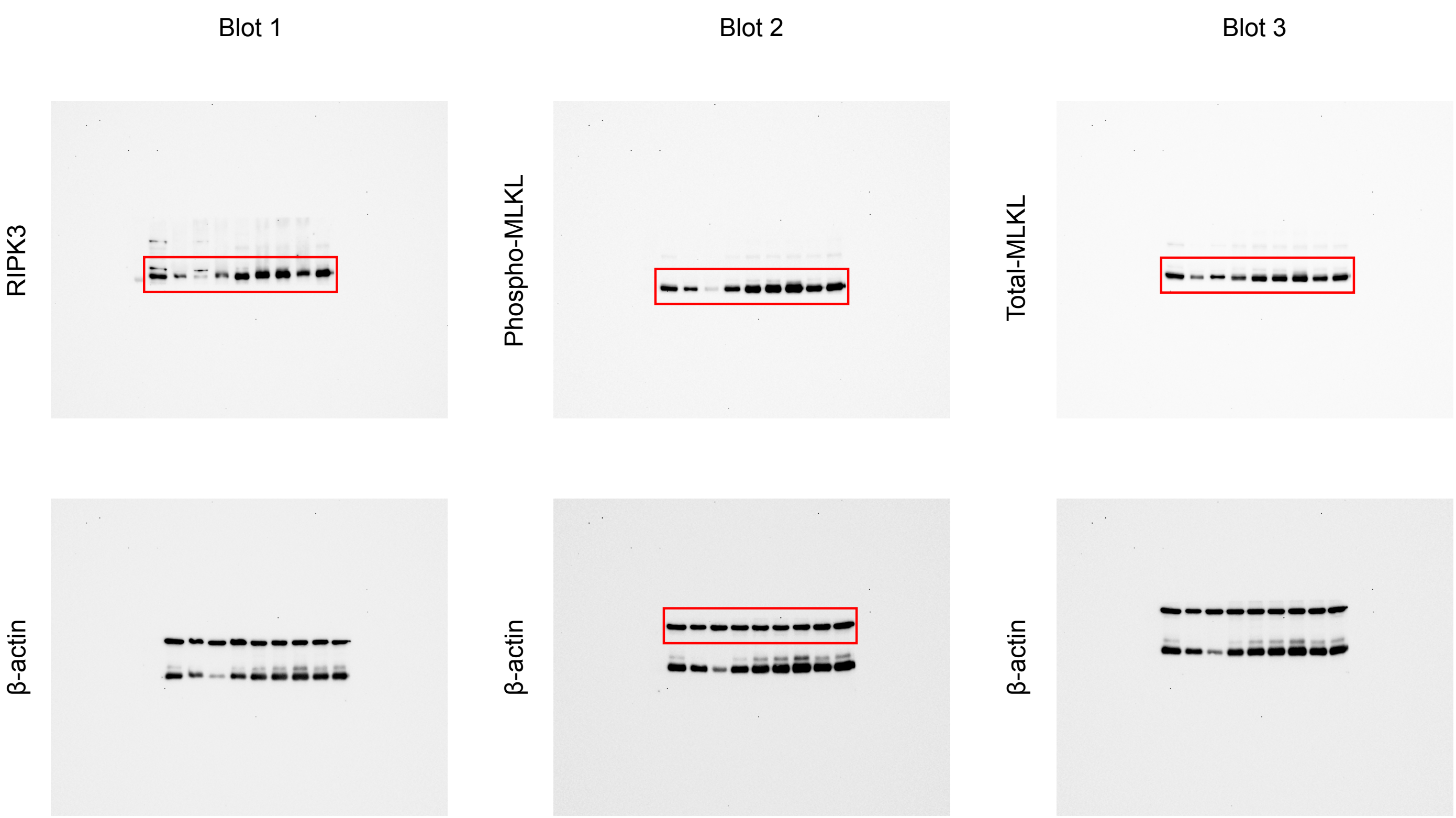
Blot 1
Blot 2
Blot 3
RIPK3
Phospho-MLKL
Total-MLKL
β-actin
β-actin
β-actin

Figure 1A

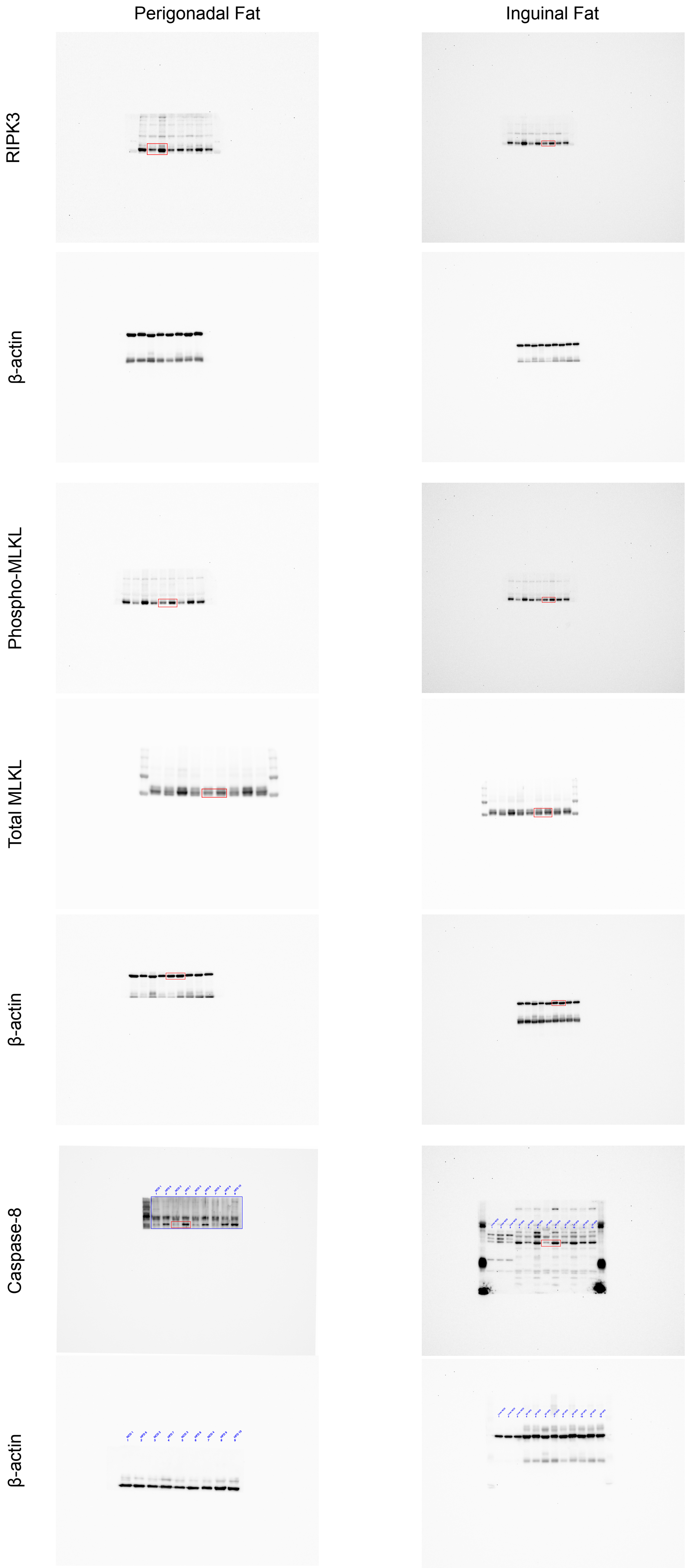

Perigonadal Fat
Inguinal Fat
RIPK3
β-actin
Phospho-MLKL
Total MLKL
β-actin
Caspase-8
β-actin

Figure 1D

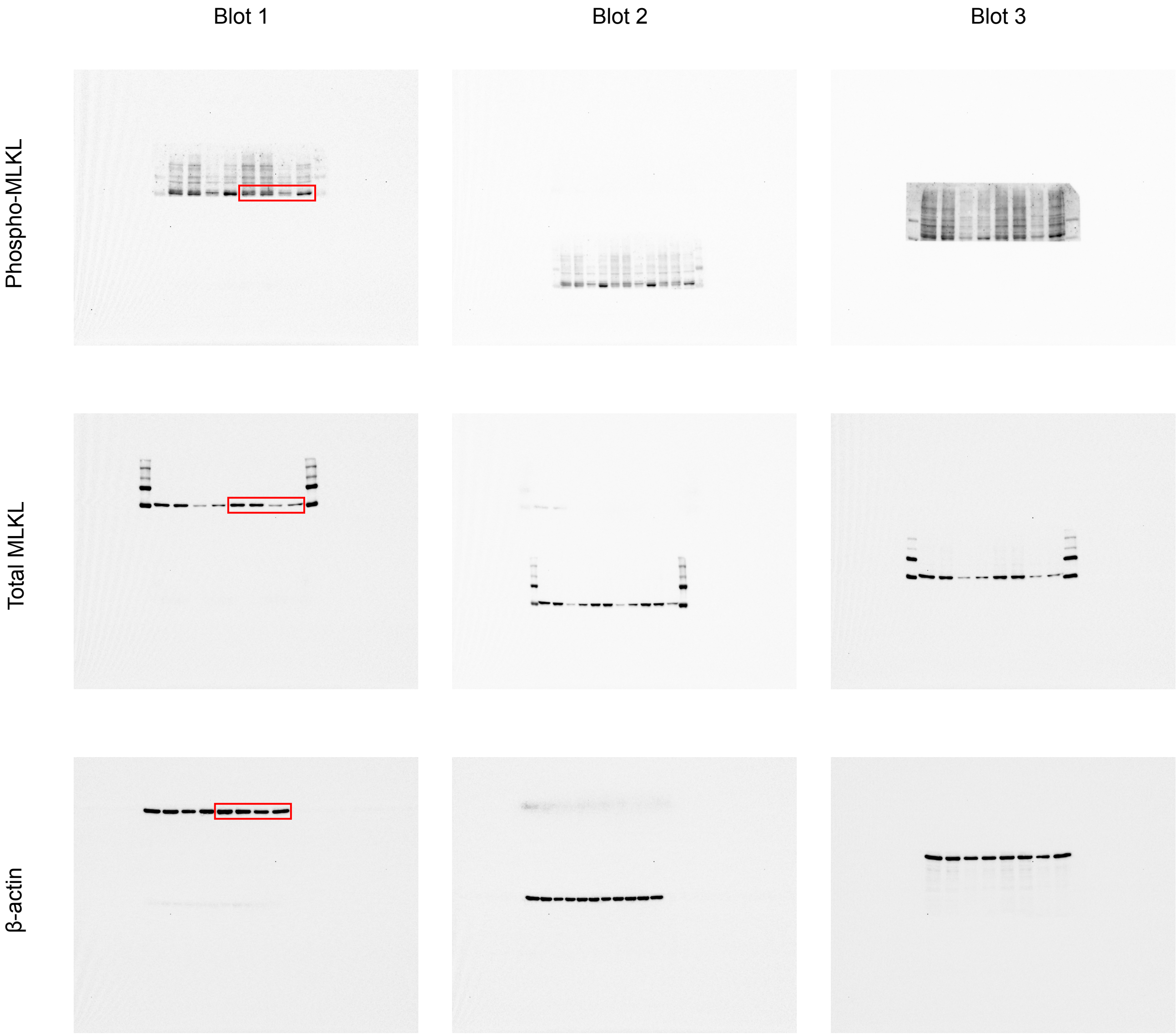

Figure 2A

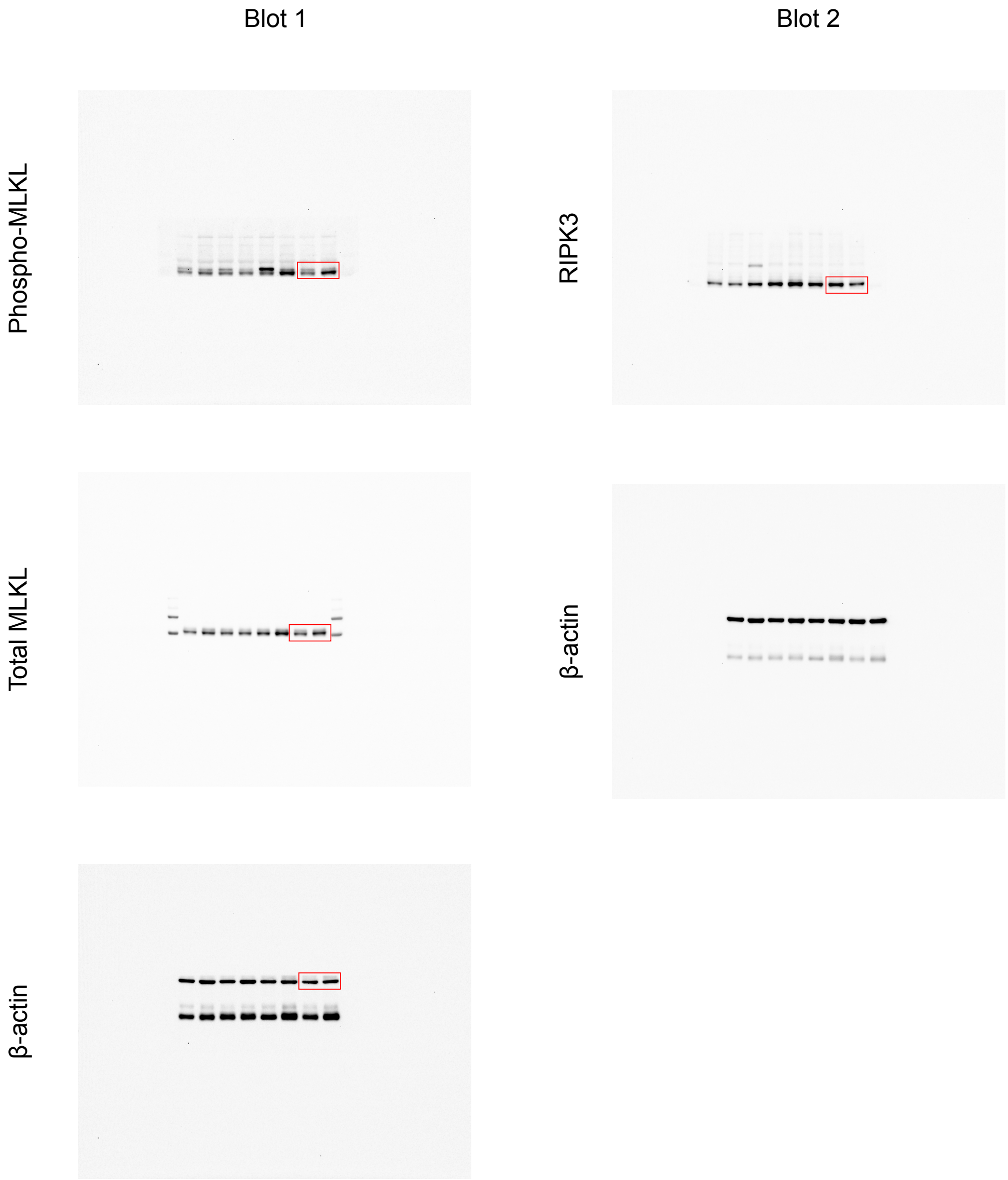

Figure 2B

RIPK3

β-actin

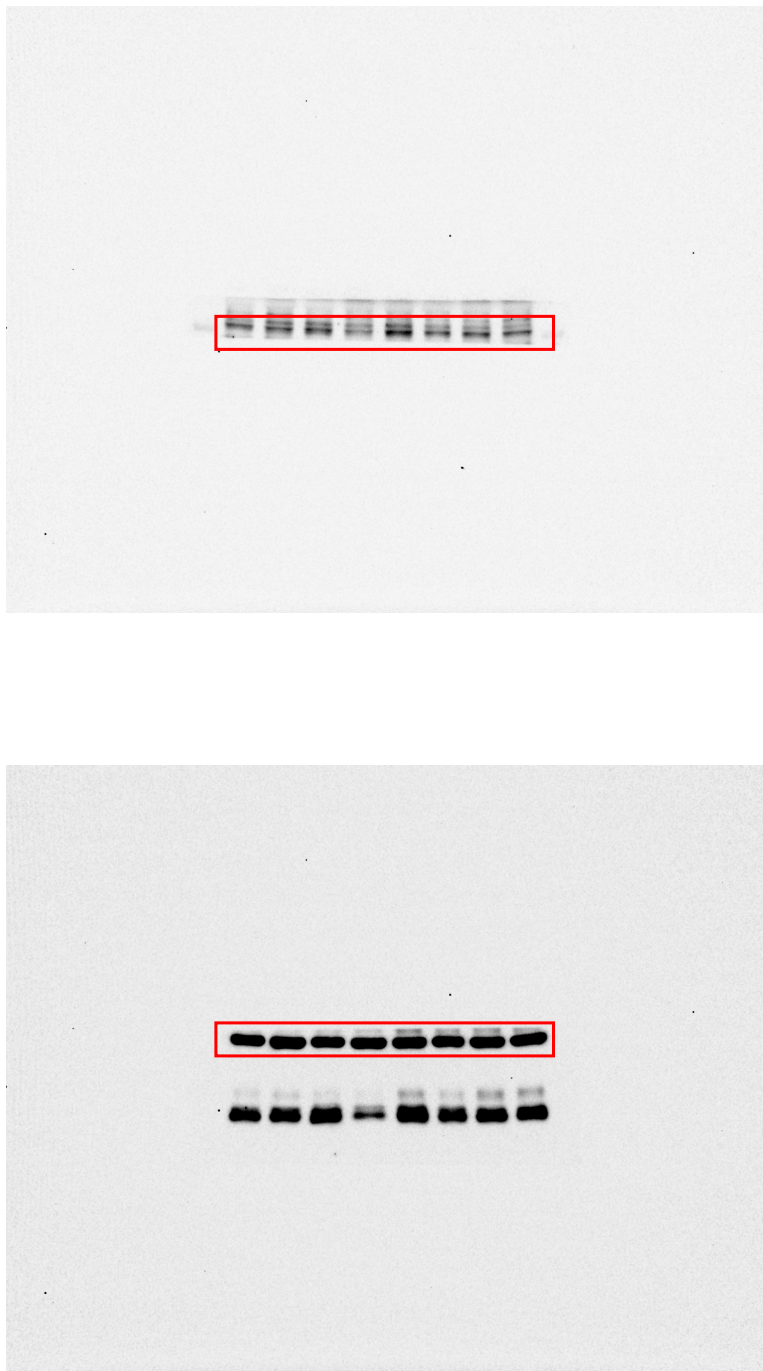

Figure 4B

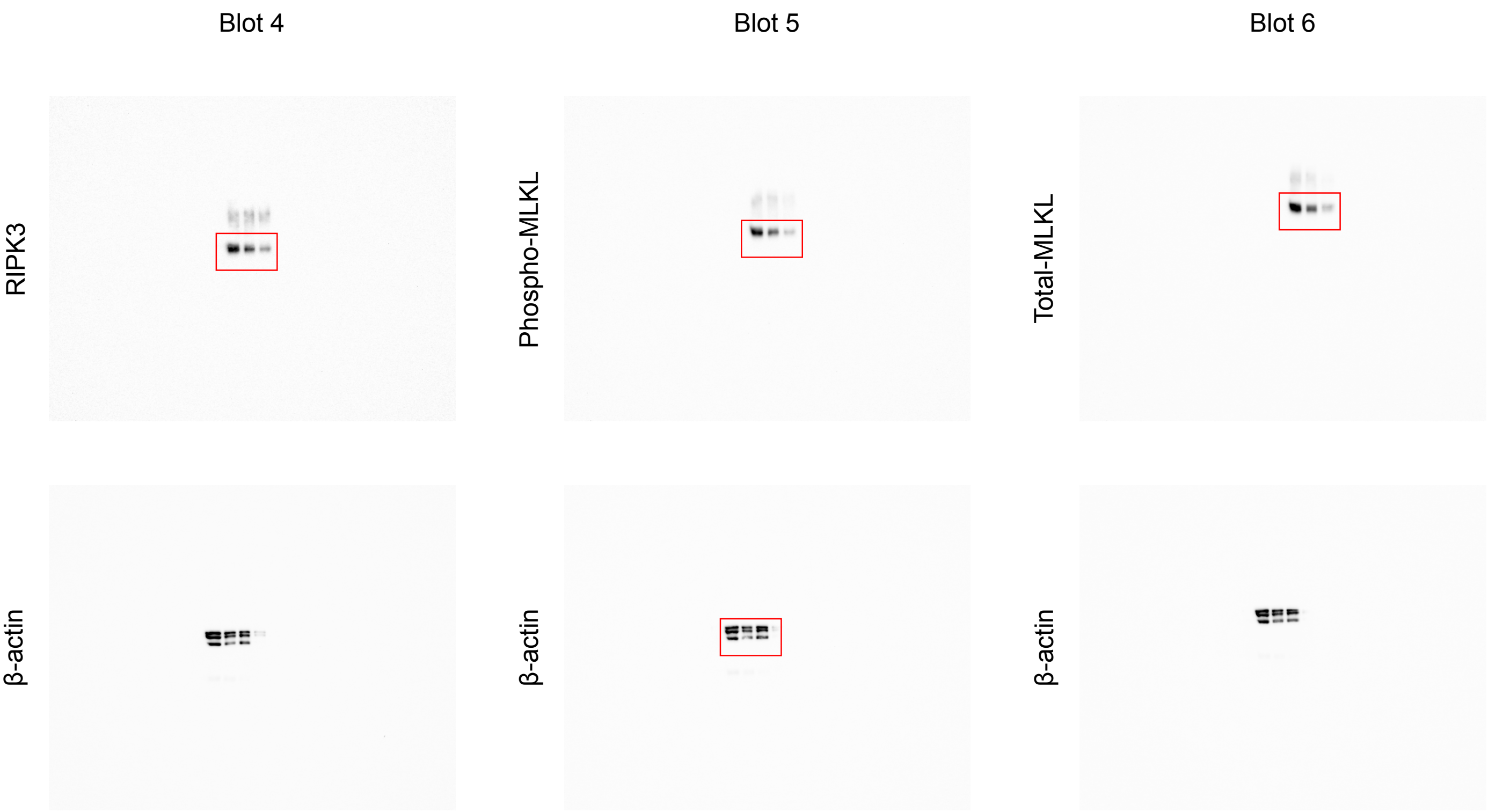

Supplementary Figure S1

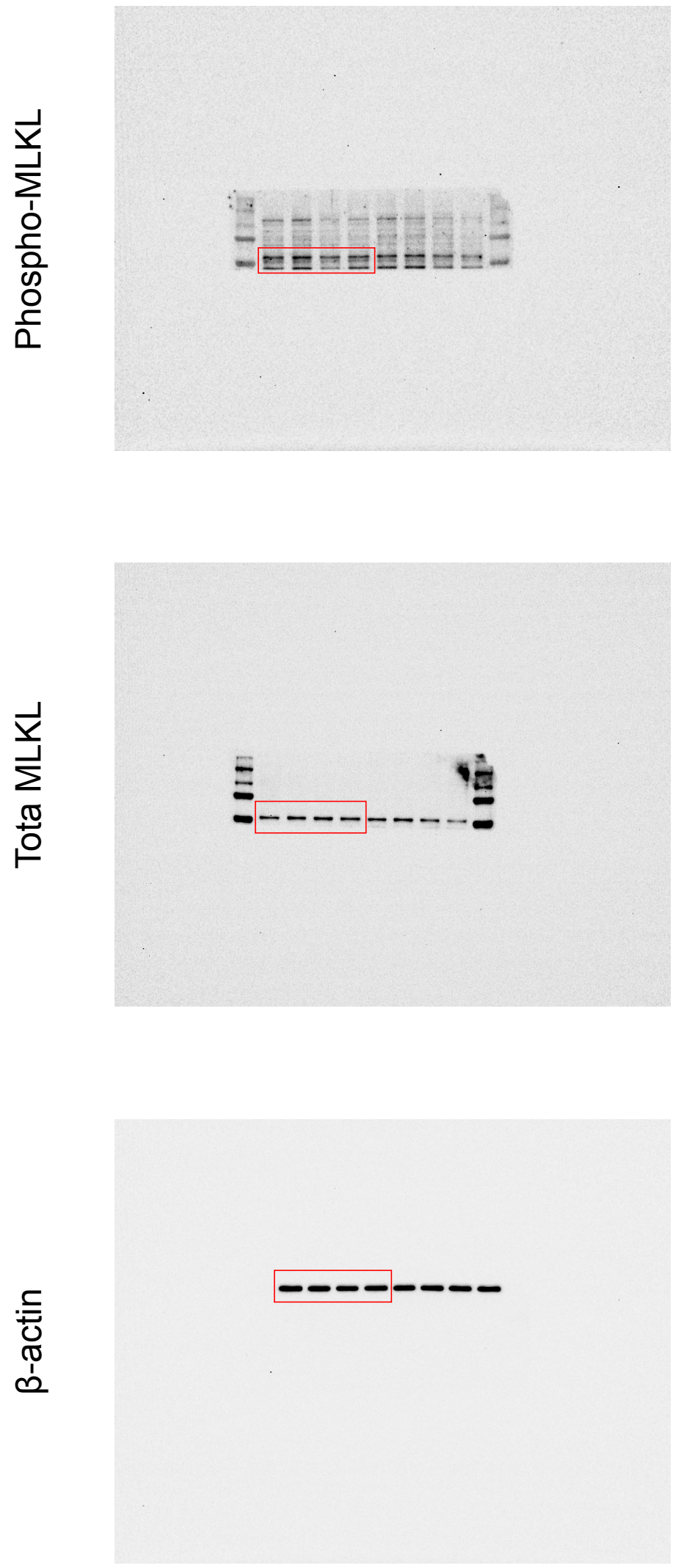

Supplementary Figure S2B

Phospho-MLKL

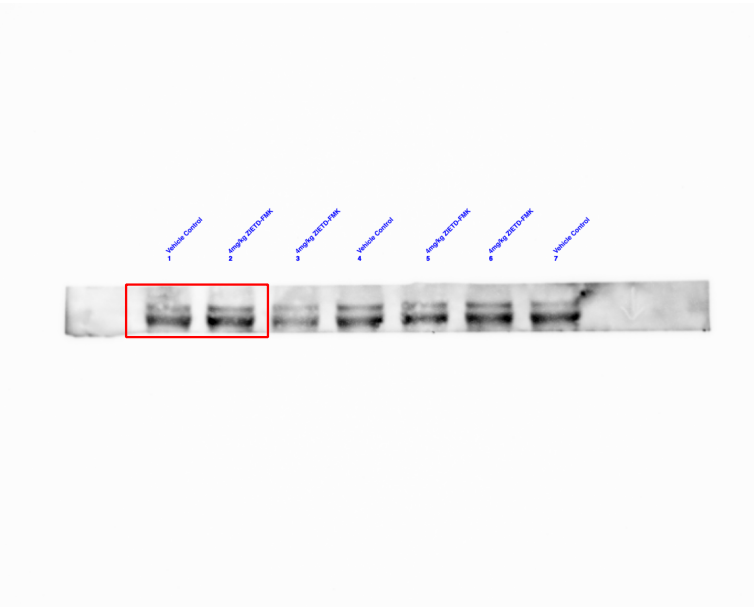

β-actin & Total-MLKL

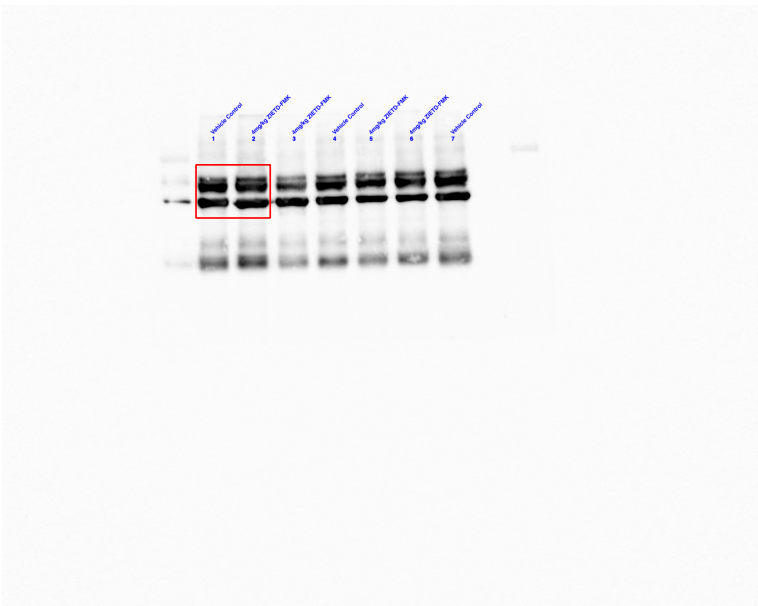

Supplementary Figure S3
