# Supplementary material for: Adipocyte caspase-8 but not RIPK3 promotes adiposity
Source: Cell Death Discov. 2026 Jun 23;12:356. doi: 10.1038/s41420-026-03201-z (PMC13547226; doi:10.1038/s41420-026-03201-z)
Supplement: Supplementary file 2 [file 41420_2026_3201_MOESM1_ESM.pdf]

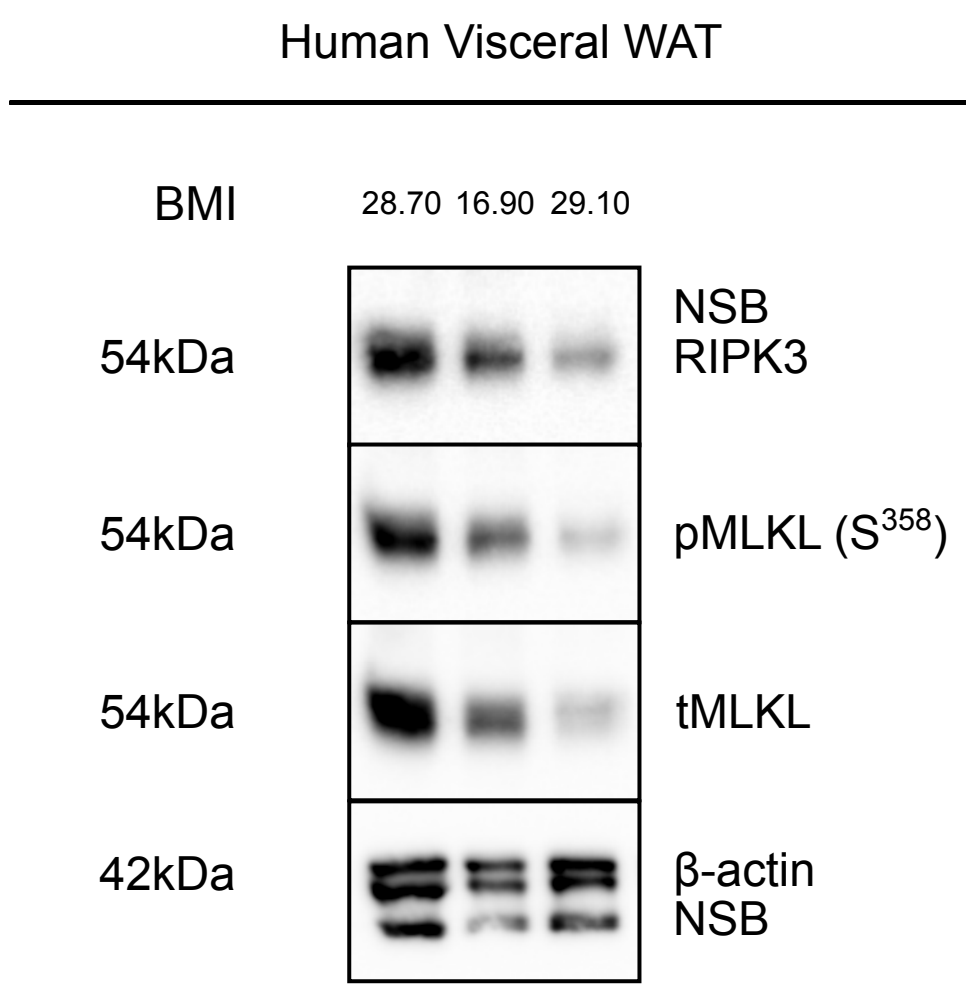

Supplementary Figure S1. RIPK3 protein expression and necroptotic signalling in additional human visceral white adipose tissue samples. Supplement to Figure 1. Immunoblot of human RIPK3, phosphorylated MLKL at S358 (pMLKL), and total MLKL (tMLKL) in human visceral WAT and body-mass index (BMI) of each subject. β-actin as loading control, representative of multiple blots. n = 3 subjects.

Supplementary Figure S1
