# Supplementary material for: Adipocyte caspase-8 but not RIPK3 promotes adiposity
Source: Cell Death Discov. 2026 Jun 23;12:356. doi: 10.1038/s41420-026-03201-z (PMC13547226; doi:10.1038/s41420-026-03201-z)
Supplement: Supplementary file 3 [file 41420_2026_3201_MOESM2_ESM.pdf]

A

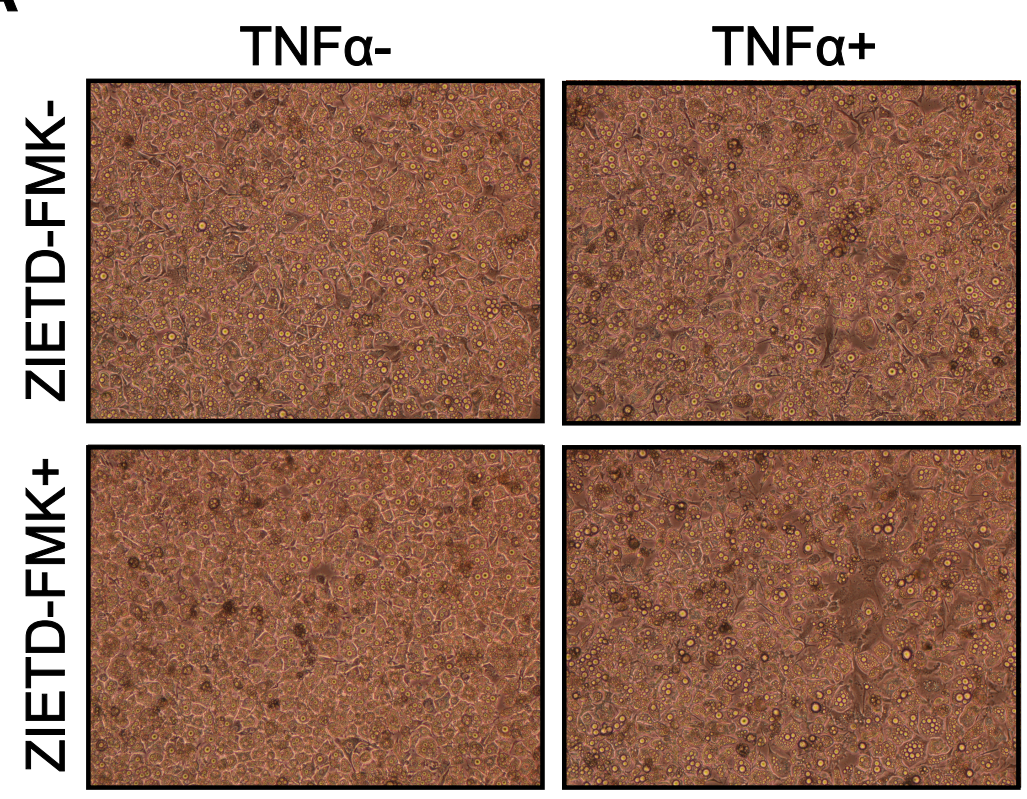

B

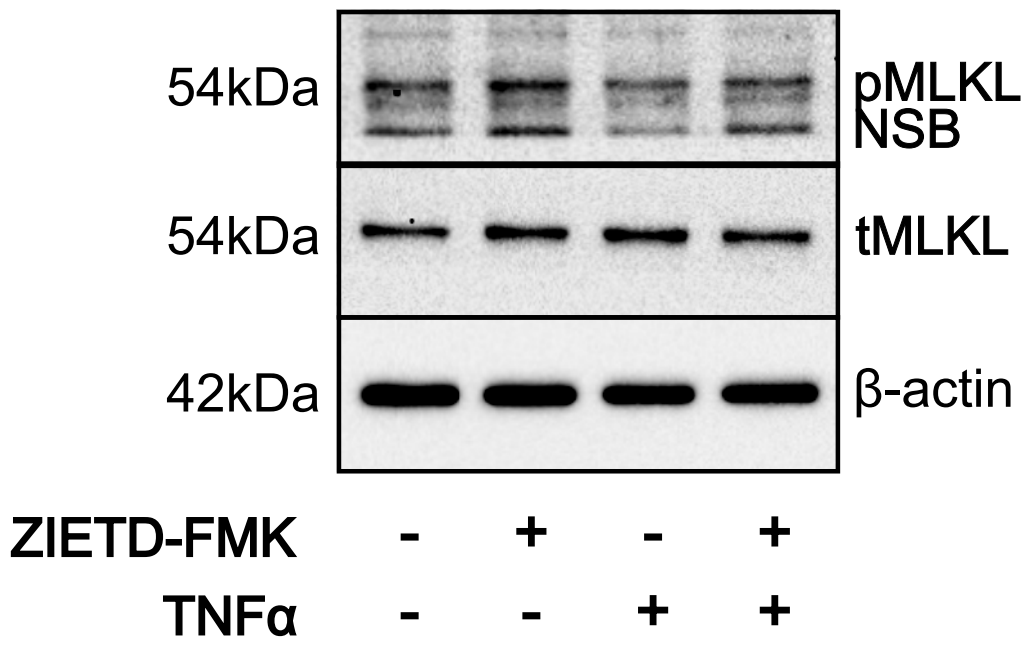

Supplementary Figure S2. TNFα alone is insufficient to induce cell death in 3T3-L1 adipocytes. **(A)** 3T3-L1 adipocytes treated with or without caspase-8 inhibitor (ZIETD-FMK) and stimulated with TNFα for a total of 24-hours. Original magnification = 10X. **(B)** Immunoblot of phosphorylated MLKL at S345 (pMLKL) and total MLKL (tMLKL) protein expression in 3T3-L1 adipocytes treated with or without caspase-8 inhibitor (ZIETD-FMK) and stimulated with TNFα for a total of 24-hours. Non-specific binding (NSB).

Supplementary Figure S2
