# Supplementary material for: Adipocyte caspase-8 but not RIPK3 promotes adiposity
Source: Cell Death Discov. 2026 Jun 23;12:356. doi: 10.1038/s41420-026-03201-z (PMC13547226; doi:10.1038/s41420-026-03201-z)
Supplement: Supplementary file 4 [file 41420_2026_3201_MOESM3_ESM.pdf]

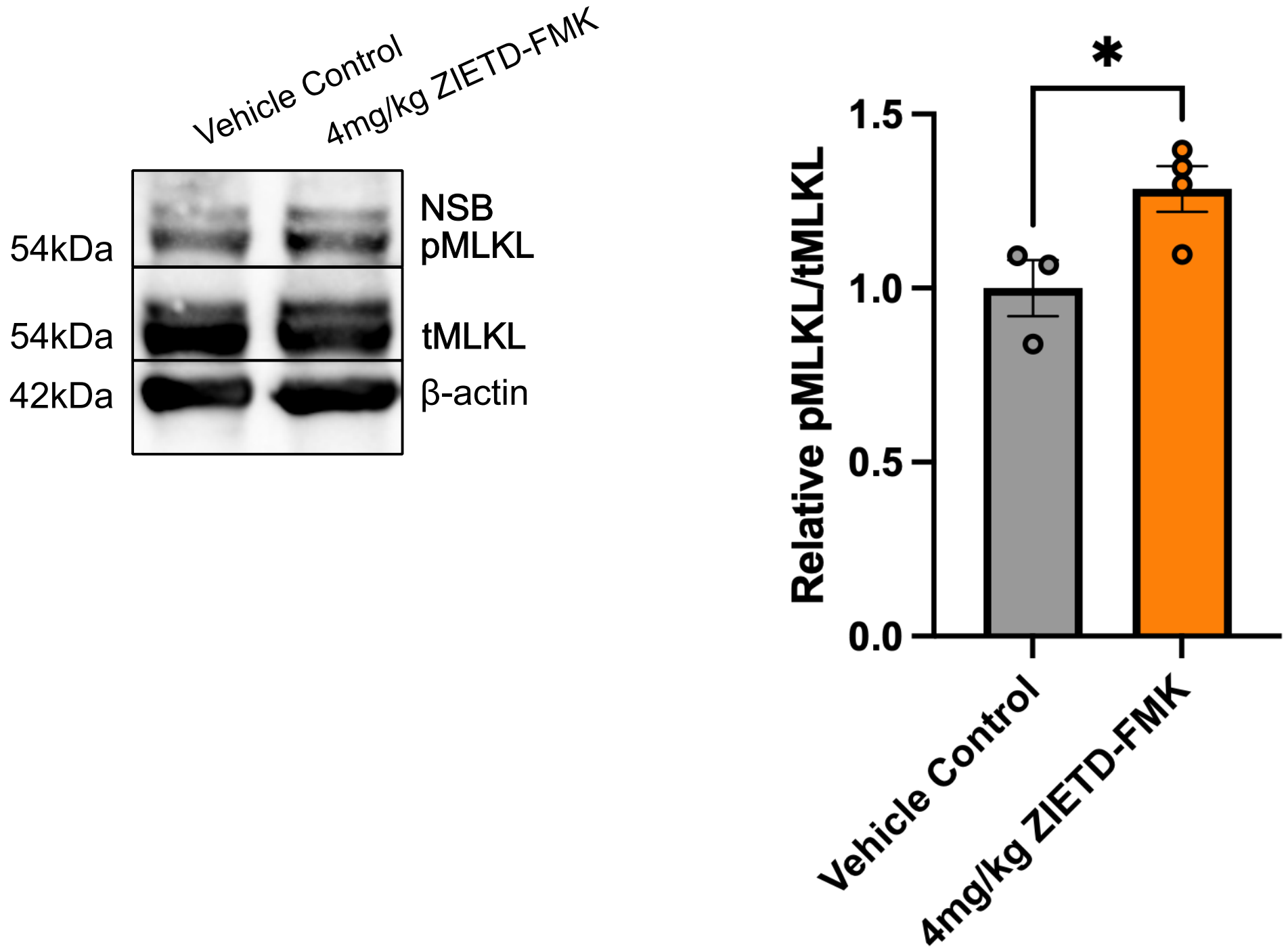

Supplementary Figure S3. Pharmacological inhibition of caspase-8 in vivo increased necroptotic signalling in white adipose tissue of mice fed HFD.

Supplement to Figure 2B. Immunoblot and quantification of phosphorylated MLKL at S345 (pMLKL) and total MLKL (tMLKL) protein expression in perigonadal WAT from mice fed 7-9 weeks of HFD starting at 6-8 weeks of age and treated with 4mg/kg caspase-8 inhibitor ZIETD-FMK ($n = 4$ male mice) or vehicle control ($n = 3$ male mice) daily for 5 days by intraperitoneal injection. β-actin as loading control. Data are mean ± SEM. * $p \leq 0.05$ by unpaired Student's *t*-test. Non-specific binding (NSB).

Supplementary Figure S3
