# Supplementary material for: Adipocyte caspase-8 but not RIPK3 promotes adiposity
Source: Cell Death Discov. 2026 Jun 23;12:356. doi: 10.1038/s41420-026-03201-z (PMC13547226; doi:10.1038/s41420-026-03201-z)
Supplement: Supplementary file 5 [file 41420_2026_3201_MOESM4_ESM.pdf]

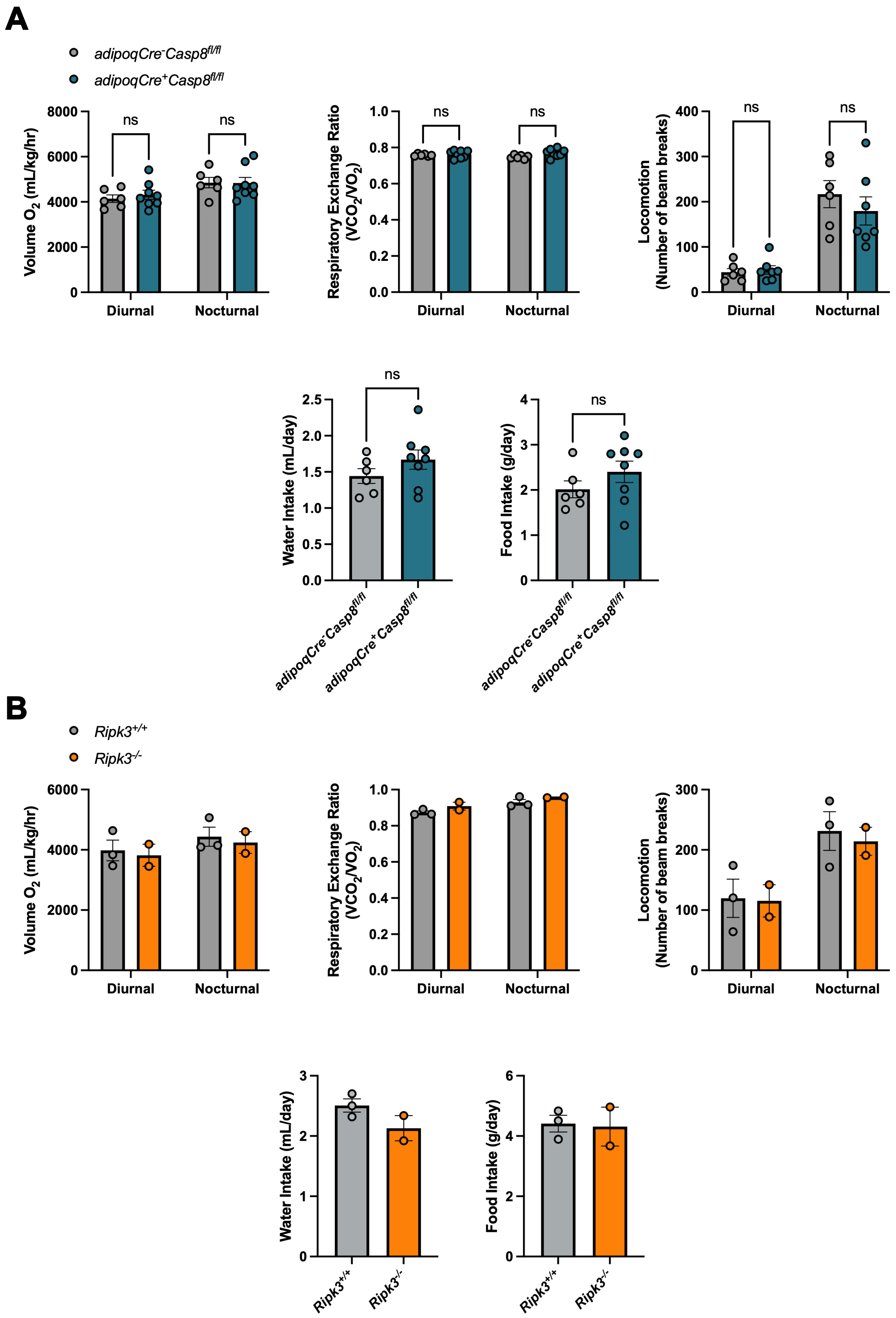

Supplementary Figure S4. Metabolic caging in mice with adipocyte-specific deletion of caspase-8 using adiponectin-Cre or global *Ripk3* deletion.

Diurnal and nocturnal energy expenditure, respiratory exchange ratio, locomotion, and water and food intake in **(A)** *adipoqCre*$^+$*Casp8*$^{fl/fl}$ and control mice fed high-fat diet (*adipoqCre*$^+$*Casp8*$^{fl/fl}$: n = 6; *adipoqCre*$^+$*Casp8*$^{fl/fl}$: n = 7-8), data are mean ± SEM. Not significant (ns) by two-way ANOVA; and **(B)** global *Ripk3*$^{-/-}$and control mice fed normal chow diet (*Ripk3*$^{+/+}$: n = 3; *Ripk3*$^{-/-}$: n = 2). Data are mean ± SEM. Not significant by Student's *t*-test.

Supplementary Figure S4
