# Supplementary material for: Adipocyte caspase-8 but not RIPK3 promotes adiposity
Source: Cell Death Discov. 2026 Jun 23;12:356. doi: 10.1038/s41420-026-03201-z (PMC13547226; doi:10.1038/s41420-026-03201-z)
Supplement: Supplementary file 6 [file 41420_2026_3201_MOESM5_ESM.pdf]

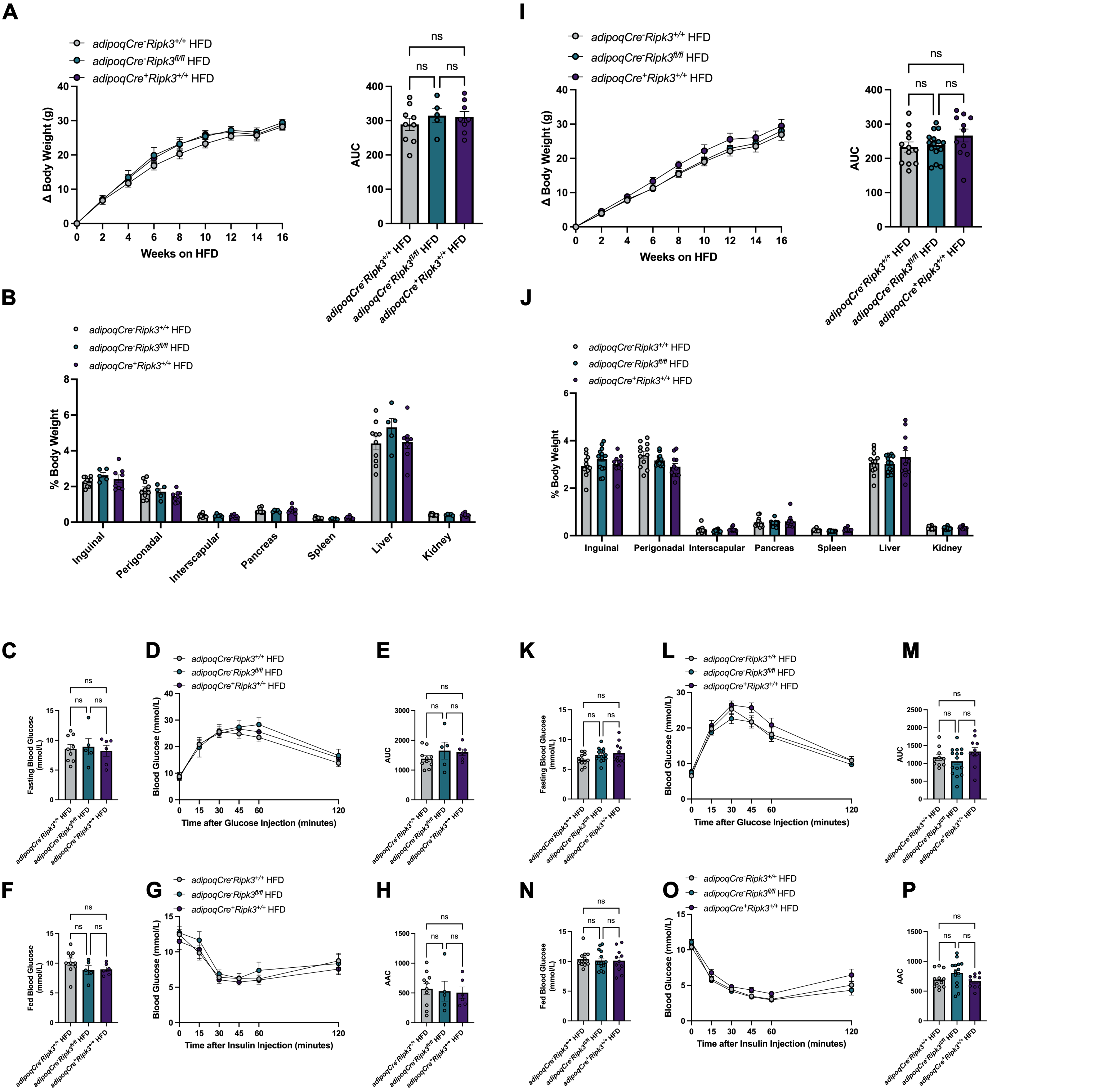

Supplementary Figure S5. No differences in obesity and glucose intolerance in littermate controls.

Total body weight (g) and area under the curve of (A) male and (I) female control mice (adipoqCre$^-$Ripk3$^{+/+}$: n = 9 males, 12 females; adipoqCre$^-$Ripk3$^{fl/fl}$: n = 5 males, 15 females; adipoqCre$^+$Ripk3$^{+/+}$: n = 8 males, 11 females) fed 60% high-fat diet (HFD) starting at 6-8 weeks of age for 16 weeks. Data are mean ± SEM. Not significant (ns) by one-way ANOVA. Tissue weights expressed as percentage of total body weight of (B) male and (J) female mice fed HFD for 16 weeks. (adipoqCre$^-$Ripk3$^{+/+}$: n = 10 males, 12 females; adipoqCre$^-$Ripk3$^{fl/fl}$: n = 5 males, 15 females; adipoqCre$^+$Ripk3$^{+/+}$: n = 8 males, 11 females). Data are mean ± SEM. Not significant (ns) by one-way ANOVA. Fasting blood glucose of (C) male and (K) female mice fed HFD for 16 weeks. (adipoqCre$^-$Ripk3$^{+/+}$: n = 9 males, 12 females; adipoqCre$^-$Ripk3$^{fl/fl}$: n = 5 males, 15 females; adipoqCre$^+$Ripk3$^{+/+}$: n = 6 males, 11 females). Data are mean ± SEM. Not significant (ns) by one-way ANOVA. Intraperitoneal glucose tolerance test (1U/kg body weight) and area under the curve (AUC) performed in (D, E) male and (L, M) female mice fed HFD for 16 weeks. (adipoqCre$^-$Ripk3$^{+/+}$: n = 10 males, 10 females; adipoqCre$^-$Ripk3$^{fl/fl}$: n = 5 males, 15 females; adipoqCre$^+$Ripk3$^{+/+}$: n = 6 males, 11 females). Data are mean ± SEM. Not significant (ns) by one-way ANOVA. Fed blood glucose of (F) male and (N) female mice fed HFD for 16 weeks. (adipoqCre$^-$Ripk3$^{+/+}$: n = 10 males, 12 females; adipoqCre$^-$Ripk3$^{fl/fl}$: n = 5 males, 15 females; adipoqCre$^+$Ripk3$^{+/+}$: n = 6 males, 11 females). Data are mean ± SEM. Not significant (ns) by one-way ANOVA. Intraperitoneal insulin tolerance test (1U/kg body weight) and area above the curve (AAC) performed in (G, H) male and (O, P) female mice fed HFD for 16 weeks. (adipoqCre$^-$Ripk3$^{+/+}$: n = 10 males, 12 females; adipoqCre$^-$Ripk3$^{fl/fl}$: n = 5 males, 14 females; adipoqCre$^+$Ripk3$^{+/+}$: n = 6 males, 10 females). Data are mean ± SEM. Not significant (ns) by one-way ANOVA.

Supplementary Figure S5
