# Supplementary material for: Adipocyte caspase-8 but not RIPK3 promotes adiposity
Source: Cell Death Discov. 2026 Jun 23;12:356. doi: 10.1038/s41420-026-03201-z (PMC13547226; doi:10.1038/s41420-026-03201-z)
Supplement: Supplementary file 7 [file 41420_2026_3201_MOESM6_ESM.pdf]

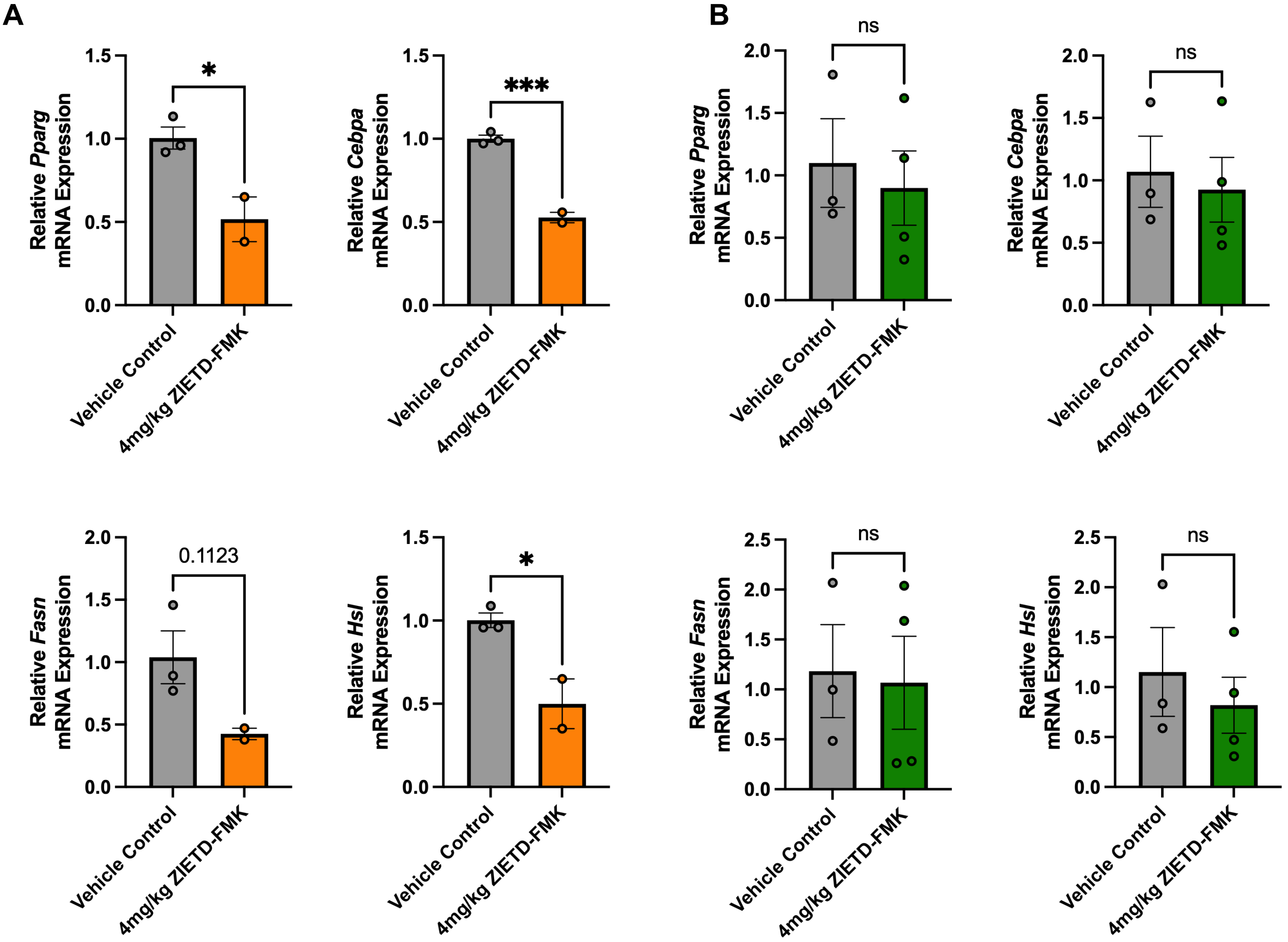

Supplementary Figure S6. Pharmacological inhibition of caspase-8 decreased adipogenic gene expression in mice fed short-term but not long-term HFD. Relative mRNA expression of key markers of mature adipocytes in mouse perigonadal WAT from mice fed HFD for **(A)** 2 weeks (Vehicle control: $n = 3$ male mice; 4mg/kg ZIETD-FMK: $n = 2$ male mice) or **(B)** 7-9 weeks (Vehicle control: $n = 3$ male mice; 4mg/kg ZIETD-FMK: $n = 4$ male mice) and treated with 4mg/kg caspase-8 inhibitor (ZIETD-FMK) or vehicle control daily for 5 days. *Bact* as endogenous control. Data are mean ± SEM. * $p \leq 0.05$, *** $p \leq 0.001$, or not significant (ns) by Student's *t*-test.

Supplementary Figure S6
